# IL-6 receptor blockade impedes proinflammatory atypical Treg subset associated with immune checkpoint inhibitor–induced inflammatory arthritis

**DOI:** 10.1172/JCI200135

**Published:** 2026-05-12

**Authors:** Yifei Ma, Nianqi Liu, Yan Li, Denghan Zhang, Shaohui He, Jun Lv, Yongluo Jiang, Guangmin Jian, Jingyao Zhang, Pengfei Zhu, Yue Ma, Jiacai Lin, Jin Li, Tong Wu, Yiwei Xu, Xiajie Lyu, Youlong Wang, Yiming Li, Yu Si Niu, Zhenyun Guo, Churong Lin, Ningnan Fang, Wei Jiang, Lihong Wang, Mengqin Yuan, Shenyue Wang, Shulin Huang, Qi Huang, Jinjian Li, Jun Lu, Bocen Chen, Guanqing Zhong, Haizhou Liu, Fadian Ding, Shangeng Weng, Rui Li, Ao Zhang

**Affiliations:** 1 Department of Hepatobiliary and Pancreatic Surgery, the First Affiliated Hospital of Fujian Medical University, Fuzhou, China.; 2 Institute of Abdominal Surgery, the First Affiliated Hospital of Fujian Medical University, Fuzhou, China.; 3 Fujian Provincial Key Laboratory of Precision Medicine for Cancer, the First Affiliated Hospital of Fujian Medical University, Fuzhou, China.; 4 Department of Hepatobiliary and Pancreatic Surgery, National Regional Medical Center Binhai Campus of the First Affiliated Hospital, Fujian Medical University, Fuzhou, China.; 5 Faculty of Psychology, Institute of Educational Science, Huazhong University of Science and Technology, Wuhan, China.; 6 Department of Pathology, State Key Laboratory of Oncology in South China, Collaborative Innovation Center for Cancer Medicine, Guangdong Key Laboratory of Nasopharyngeal Carcinoma Diagnosis and Therapy, Sun Yat-sen University Cancer Center, Guangzhou, China.; 7 State Key Laboratory of Oncology in South China, Guangdong Key Laboratory of Nasopharyngeal Carcinoma Diagnosis and Therapy, Guangdong Provincial Clinical Research Center for Cancer, Sun Yat-sen University Cancer Center, Guangzhou, China.; 8 Spinal Tumor Center, Department of Orthopaedic Oncology, Changzheng Hospital, Naval Medical University, Shanghai, China.; 9 Department of Infectious Diseases, the First Affiliated Hospital of Zhengzhou University, Zhengzhou, China.; 10 Department of Nuclear Medicine, Sun Yat-sen University Cancer Center, Guangzhou, China.; 11 Department of Clinical Laboratory & Key Clinical Laboratory of Henan Province, The First Affiliated Hospital of Zhengzhou University, Zhengzhou, China.; 12 Department of Medicine, Nuvance Health Danbury Hospital, Danbury, Connecticut, USA.; 13 Department of Laboratory Medicine, Taiyuan Central Hospital of Shanxi Medical University, Taiyuan, China.; 14 Department of Neurology, Hainan Hospital of Chinese PLA General Hospital, Sanya, China.; 15 Department of Anesthesiology, Shanxi Bethune Hospital, Shanxi Academy of Medical Sciences, Third Hospital of Shanxi Medical University, Tongji Shanxi Hospital, Taiyuan, China.; 16 Department of Ophthalmology, Hainan Hospital of Chinese PLA General Hospital, Sanya, China.; 17 Department of Clinical Laboratory Medicine, Cancer Hospital of Shantou University Medical College, Shantou, China.; 18 Internal Medicine Department, Jacobi Medical Center 1400 Pelham Play S, Bronx, New York, USA.; 19 Department of General Surgery, Hainan Hospital of Chinese PLA General Hospital, Sanya, China.; 20 Department of Neurosurgery, Beijing Tiantan Hospital, Capital Medical University, Beijing, China.; 21 Acute Communicable Disease Epidemiology Division, Dallas County Health and Human Services, Dallas, Texas, USA.; 22 Department of Radiology, the Third Affiliated Hospital of Sun Yat-Sen University, Guangzhou, China.; 23 Department of Orthopedics and Spine Surgery, Cancer Hospital of Shantou University Medical College, Shantou, China.; 24 National Regional Medical Center, Binhai Campus of the First Affiliated Hospital, Fujian Medical University, Fuzhou, China.; 25 Department of Bioinformatics, Fujian Key Laboratory of Medical Bioinformatics, School of Medical Technology and Engineering, Fujian Medical University, Fuzhou, China.; 26 Department of Neurology of First Affiliated Hospital, Fujian Medical University, Fuzhou, China.; 27 Institute of Neuroscience, Fujian Medical University, Fuzhou, China.; 28 School of Basic Medical Sciences, Fujian Medical University, Fuzhou, China.; 29 Department of Clinical Laboratory, State Key Laboratory of Oncology in South China, Collaborative Innovation Center for Cancer Medicine, Guangdong Key Laboratory of Nasopharyngeal Carcinoma Diagnosis and Therapy, Sun Yat-sen University Cancer Center, Guangzhou, China.; 30 Department of Hepatobiliary Surgery, the First Affiliated Hospital of Shantou University Medical College, Shantou, China.; 31 Key Laboratory of Biochemistry and Molecular Biology, Hainan Medical University, Haikou, China.

**Keywords:** Immunology, Oncology, Cancer immunotherapy

## Abstract

**BACKGROUND:**

Immune checkpoint inhibitor–induced inflammatory arthritis (ICI-IA) significantly impairs cancer therapy and patient quality of life, yet its pathogenic mechanisms remain unclear.

**METHODS:**

Through integrated single-cell multi-omics analysis of paired peripheral blood, synovial fluid, and tumor samples from longitudinal ICI-IA cohorts and matched controls, we identified a unique regulatory T-cell (Treg) population coexpressing CD137 and IL-6R (AtpTreg).

**RESULTS:**

These cells exhibited reduced immunosuppressive capacity while aberrantly producing high levels of IL-17 and promoting proinflammatory responses of synoviocytes. AtpTreg exhibits shared clonotypes and phenotypes across tissue compartments. Notably, AtpTreg frequency correlates with increased arthritis severity yet paradoxically associates with improved overall survival. Anti-IL6R therapy reduced AtpTreg levels, corresponding with improved arthritis outcomes and quality of life, without compromising anti-tumor immunity.

**CONCLUSION:**

Our findings define a pathogenic Treg subset in ICI-IA and validate IL-6R blockade as a mechanism-based therapeutic strategy, bridging mechanistic discovery to clinical translation.

**TRIAL REGISTRATION:**

NCT07357636.

**FUNDING:**

The National Natural Science Foundation of China General Fund Project; National Natural Science Foundation of China Youth Science Fund Project; Regional joint key support project of National Natural Science Foundation of China; Natural Science Foundation of Fujian Province; Joint Funds for the Innovation of Science and Technology, Fujian Province.

## Abstract 2

A newly discovered Treg cell causes immunotherapy-induced arthritis; blocking IL-6R treats it safely without hurting anti-tumor immunity

## Graphical abstract

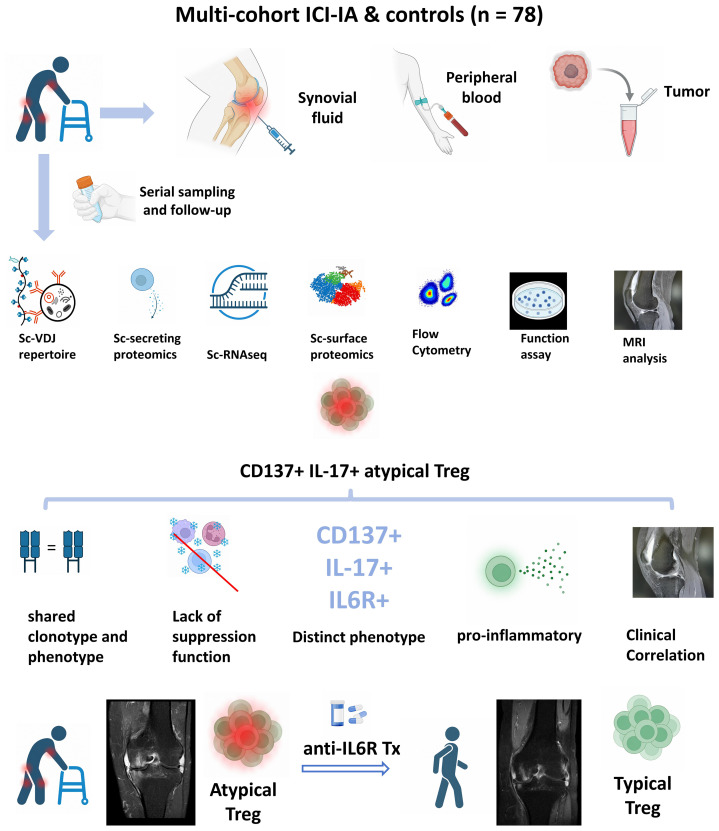

## Introduction

Immune checkpoint inhibitors (ICIs) have transformed oncology by unleashing antitumor immunity, yet their efficacy is often limited by immune-related adverse events (irAEs) (1). Among these, Immune checkpoint inhibitor–induced inflammatory arthritis (ICI-IA) poses a significant clinical challenge, causing persistent joint damage and functional decline that can compromise both long-term quality of life and cancer treatment continuity (2–4). Despite its growing recognition, the immunopathology of ICI-IA remains poorly understood, particularly in how systemic immune dysregulation intersects with localized tissue injury (4–6). Unlike classical autoimmune arthritis, ICI-IA emerges in the context of cancer immunotherapy, suggesting a unique interplay between antitumor immunity and loss of self tolerance. Unraveling these mechanisms is critical not only for mitigating irAEs but also for elucidating potential links between autoimmunity and treatment efficacy — a paradox observed in clinical cohorts, but, remains mechanistically unresolved (7–9).

Central to this puzzle is the role of regulatory T cells (Tregs), which typically restrain excessive immune activation (10). While ICIs are thought to reduce systemic Treg suppression, their association with ICI-IA implies a more complex, tissue-specific dysfunction (11). Whether pathogenic Treg subsets arise during ICI therapy, how they contribute to joint inflammation, and whether they simultaneously reflect heightened antitumor immunity are open questions (12). Addressing these gaps requires a multidimensional approach, combining deep immune profiling with longitudinal clinical data to uncover actionable therapeutic targets. Such insights could pave the way for precision management of ICI-IA — balancing irAE control with preservation of anticancer immunity.

To bridge these gaps, we conducted a translational study integrating single-cell multi-omics with paired tissue analyses and clinical outcomes. By profiling peripheral blood, synovial fluid, and tumor samples from ICI-treated patients across multiple cohorts, we aimed to identify disease-defining immune subsets and trace their clonal dynamics over time. Crucially, we linked these findings to arthritis progression and cancer survival, exploring whether specific immune signatures could inform clinical decision making. Beyond mechanistic insights, we sought to evaluate a targeted therapeutic strategy derived from these observations, testing its impact on both ICI-IA symptoms and underlying immune pathology. Our work not only delineates the cellular basis of ICI-IA but also provides a framework for translating immune discoveries into tailored therapies — addressing an urgent need in the growing population of cancer survivors living with irAEs.

## Results

### Identifying an atypical Treg cell type as a signature of ICI-IA after checkpoint blockade.

**Figure 1:**
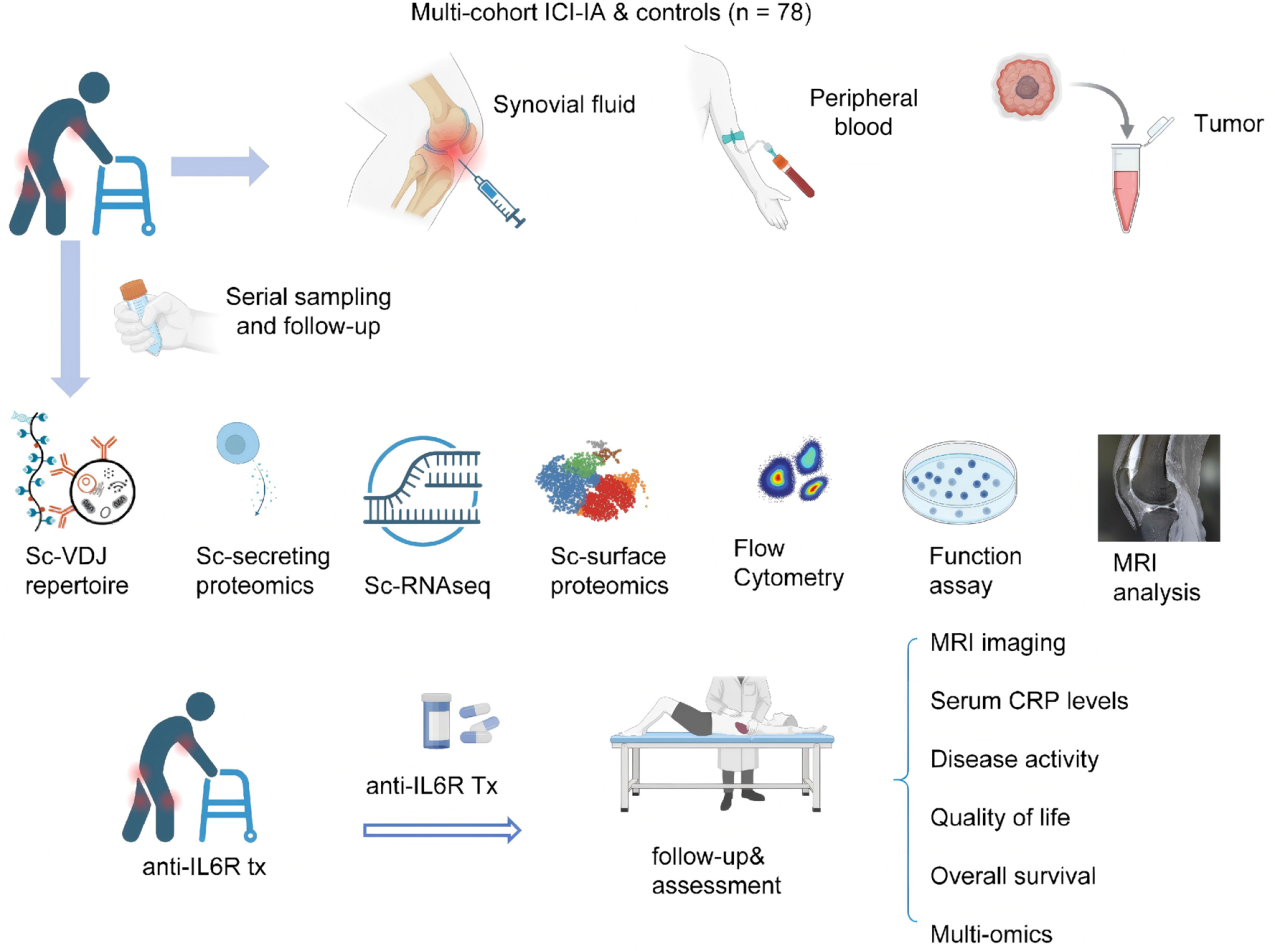
Study design and multi-omics workflow. Schematic overview of the study design, patient enrollment, sample collection, and multi-omics analyses. A total of 78 participants were enrolled across multiple centers. Longitudinal paired samples from peripheral blood, synovial fluid (SF), and tumor tissue were collected at multiple time points (ICI-naive, preclinical, disease onset, and follow up). These samples were subjected to integrated single-cell multi-omics analyses. An independent cohort of ICI-IA patients treated with anti–IL-6 receptor therapy was enrolled, with paired blood and SF samples collected before and after treatment for comparative analysis. Concurrent longitudinal clinical assessments were performed to correlate immune phenotypes with clinical outcomes.

**Figure 2:**
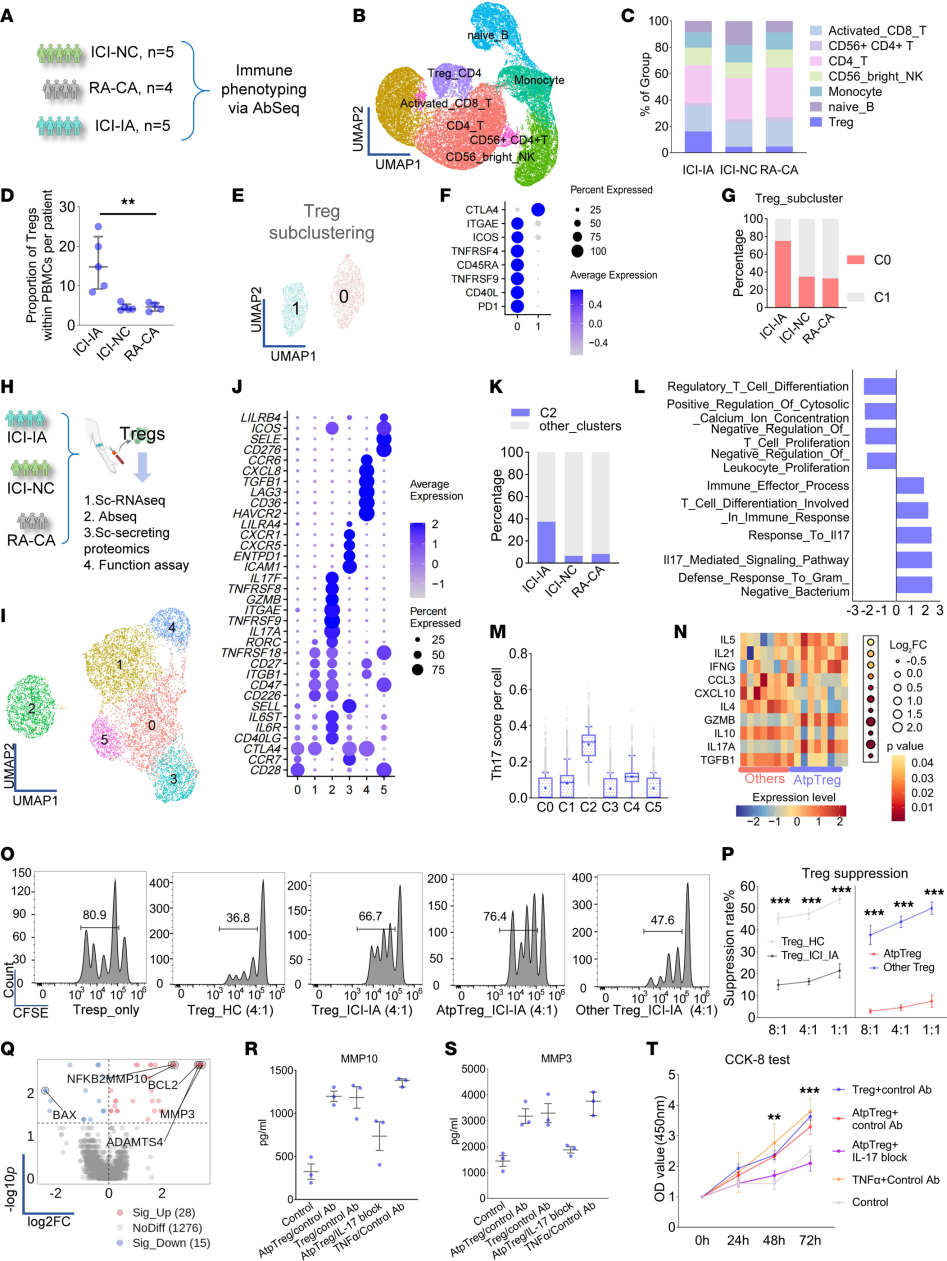
An atypical Treg cell type as signature of inflammatory arthritis after checkpoint blockade. (**A**) Research flowchart of single-cell surface proteomics of peripheral blood mononuclear cells from ICI-IA (17,323 cells, *n* = 5), RA-CA (17,541 cells, *n* = 4) and ICI-NC (18,619 cells, *n* = 5). (**B**) Annotated UMAP clustering defined by single-cell surface proteomics of blood samples of ICI-IA, RA-CA, and ICI-NC. (**C**) Distribution of cell clusters in each group. (**D**) Distribution of Treg cells per patient in each group. (**E**) UMAP subclustering of Treg cells (3,744 cells) from the original single-cell surface proteomics samples. (**F**) Signature proteins of the 2 Treg subclusters depicted as the bubble plot. (**G**) Distribution of Treg subclusters in each group, showing ICI-IA group has high percentage cluster # 0. (**H**) Research flowchart of single-cell RNA-seq (11,864 cells) of bead-enriched Treg cells. (**I**) UMAP clustering defined by single-cell RNA-seq of blood Treg cells of all samples. (**J**) Signature proteins. (**K**) Distribution of single-cell RNA-seq–defined cell clusters in each group. (**L**) GSEA of differential genes in cluster # 2. (**M**) Th17 scoring in each cluster. (**N**) Heatmap of average expression of each marker of single-cell secreting proteomics of AtpTreg and other Tregs per sample. (**O** and **P**) Treg suppression assay with Tregs for Tresp from healthy controls. FlowJo figures are shown for CFSE negative cells at a Tresp‑to‑Treg ratio of 1:4. (**O**) and suppression analysis plot shown (**P**). (**Q**) OLINK proteomics of human synovial lines (MH7A) cocultured with blood-derived Treg cells of patients with ICI-IA, as compared with human synovial lines (MH7A) without coculture system. (**R** and **S**) ELISA test of stromelysin MMP-10 (**R**) and stromelysin MMP-3 (**S**) in MH7A in coculture system. (**T**) CCK-8 test of cell counts of MH7A in coculture system. **P < 0.01, ***P < 0.001, calculated using an independent Wilcoxon test. *P < 0.05, **P < 0.01, ***P < 0.001, calculated with independent Wilcoxon signed-rank test.

To profile the cellular phenotype of patients with ICI-IA, we obtained CD45^+^ PBMC samples (Supplemental Table 1, Figure 1, and Figure 2A; supplemental material available online with this article; https://doi.org/10.1172/JCI200135DS1) of patients with ICI-IA at onset (*n* = 5; 17,323 cells) as well as 2 control groups, including rheumatoid arthritis comorbid with cancer (RA-CA, *n* = 4; 17,541 cells), as well as ICI-treated non-irAE cancer patients (ICI-NC, *n* = 5; 18,619 cells). We first examined the cell-surface phenotype using single-cell surface proteomics (Abseq 37-antibody panel in Supplemental Files). UMAP reduction of a panel of 37 markers identified 7 cell clusters (Figure 2B and Supplemental Figure 1A), in which CD25^+^ CTLA4^+^ Tregs (Figure 2C and Supplemental Figure 1B) were significantly increased in patients with ICI-IA while other cell types were not different among the 3 groups (Figure 2D). This finding is in contrast with previous studies that reported that ICI-induced systemic immunity usually involves Treg cell depletion (11, 13–15). This divergence may reflect immune alterations specific to irAE development, where checkpoint blockade paradoxically expands pathogenic Treg subsets in patients who are susceptible. Subclustering analysis further revealed that Tregs of patients with ICI-IA exhibited an activated memory type (ICOS^+^, CD40L^+^, PD1^+^, and CD137^+^, cluster # 0) with lower expression of inhibitory markers (e.g., CTLA-4, HAVCR2, LAG3, Figure 2, E–G). These initial findings identify a distinct expanded population of Tregs with an atypical pathogenic phenotype (AtpTreg) in patients with ICI-IA.

Considering the classic role of Treg cells in maintenance of immune tolerance and checking of autoimmune reactions, the atypical finding of Treg phenotype prompted further investigation at single-cell RNA-seq level (Supplemental Table 1 and Figure 2H). We isolated CD4^+^CD25^+^CD127^–^ cells from ICI-IA (*n* = 4), RA-CA (*n* = 3), and ICI-NC (*n* = 4). A total of 11,864 cells spanning 9,364 genes were identified, with UMAP reduction into 6 clusters (Figure 2I). Consistent with findings in Abseq, this activated Treg cluster (cluster # 2, named atypical Treg, AtpTreg) was specific to patients with ICI-IA as compared with controls (Figure 2, J and K). While AtpTreg does not express classic inhibitory molecules such as *CTLA4* and *LAG3*, it exhibits a cytotoxic Th17-like phenotype (*TNFRSF9*^+^ *CCR7*^–^, *IL17A*^+^, *GZMB*^+^, *RORC*^+^, *EMOES*^+^). CD25 (*IL2RA*) was broadly expressed across all Treg clusters and thus is not featured in the DEG bubble plot; its expression is shown in Supplemental Figure 1E. Notably, AtpTregs maintain high levels of *FOXP3* and IL-2RA expression (Supplemental Figure 1C). A biological annotation pathway of these altered genes pointed to inflammatory pathology (Figure 2L) and IL-17-related pathway. Consistently, Th17 scores were significantly higher in AtpTreg (Figure 2M). Pseudotime analysis further reveals that the phenotype of AtpTreg is featured by coexpression of *RORC* and *EOMES* (Supplemental Figure 1, D and E).

Next, we further interrogated the functional profile of AtpTreg. Given that CD137 is uniquely expressed by AtpTreg, we isolated AtpTreg cells from 8 patients with ICI-IA at disease onset based on their expression of CD137 (Figure 2B). We performed single-cell surface proteomics (Abseq 35-antibody panel) as well as single-cell secreting proteomics (Isoplexis 32-secreted protein chip) on AtpTreg and other Treg cells (Figure 2O). We confirmed our scRNAseq data showing that CD137^+^ AtpTreg express higher levels of CD137, PD1, ICOS, CD40L, and IL-6R (Supplemental Figure 1, F–H). We also observed that CD137^+^ AtpTreg secrete increased levels of IL-17A and Granzyme B (Figure 2N and Supplemental Figure 1I) compared with CD137^–^ Treg. Secretion of IL-4, IL-10 and TGFβ was reduced. Together, these findings suggested that AtpTreg is a unique feature of ICI-IA.

Then, we queried whether AtpTreg would be pathogenic in in vitro settings. We performed flow cytometry–based suppression assay to assess in-vitro Treg inhibition of responder T cell (Tresp) proliferation from healthy control donor peripheral blood (HC). Treg suppression assay was performed with Tregs in each group for T responder (Tresp) from healthy controls. CFSE-labelled Tresp cells were cocultured with Tregs from healthy controls (Tregs_HC), Tregs from patients with ICI-IA (Tregs_ICI-IA), AtpTregs (ICI-IA), and other Tregs (ICI-IA). Overall, Tregs of blood from patients with ICI-IA showed significantly decreased ability to inhibit HC Tresp proliferation compared with healthy controls (Figure 2, O and P, with gating strategy shown in Supplemental Figure 1, J and K). We then isolated AtpTregs from patients with ICI-IA and compared the inhibition ability with other Treg cells and found that AtpTreg cells exhibited significantly decreased suppression function than other Treg cells. In a preliminary assay, we also observed a trend where Tregs from patients with ICI-IA and particularly the CD137^+^ subset appeared less suppressive of TNF-α secretion by Tresp cells (Supplemental Figure 9). These findings primarily indicated dysfunctional AtpTreg cells of patients with ICI-IA (Figure 2, O and P). To test whether Treg cells would be associated with synovial inflammation, we cocultured Tregs with human synovial cell lines ‌MH7A (Figure 2, Q–T). We demonstrated that Tregs of ICI-IA patient peripheral blood cause increased expression of synovial inflammation proteins by means of OLINK proteomics (Figure 2Q) and 2 marker proteins (metalloproteinases, MMP3, MMP10) of articular damage increased by coculture with AtpTreg cells (Figure 2, R and S). Notably, AtpTreg cells can increase proliferation of synovial cell lines, and, interestingly, IL-17 blocker can diminish the expression of MMP3/10 as well as cell proliferation (Figure 2, R and S). Combined with previous data on AtpTreg cell phenotype, these data suggested that the ICI-IA–associated AtpTregs exhibit loss of regulatory functions and cause articular damage.

### Stable AtpTreg cell phenotype and clonotype sharing across blood, synovial fluid, and tumor samples in patients with ICI-IA.

**Figure 3:**
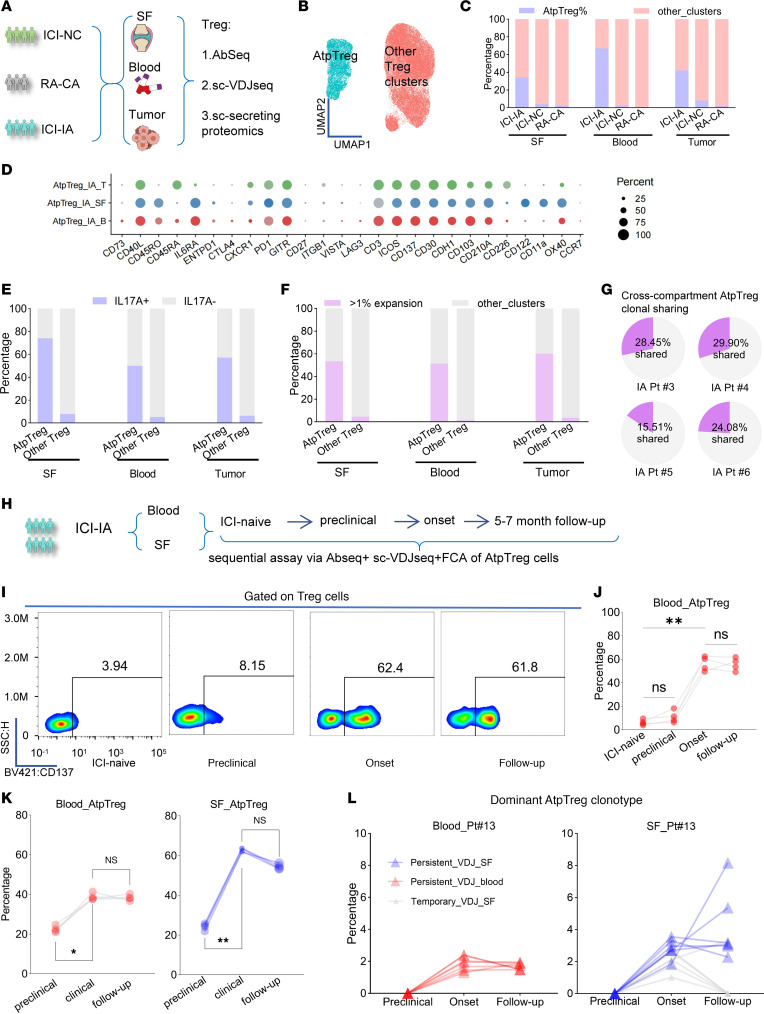
Stable AtpTreg cell phenotype and clonotype across blood, synovial fluid, and tumor samples. (**A**) Research flowchart showing grouping of bead-enriched Treg cells from paired samples. (**B**) UMAP clustering annotated by single-cell surface proteomics. (**C**) AtpTreg cell percentage in each group and compartment sample, showing that ICI-IA group has high AtpTreg population in blood, SF, and tumor samples. (**D**) Signature proteins of AtpTreg clusters in blood, SF, and tumor samples of patients with ICI-IA, depicted as the bubble plot. (**E**) IL17A-secreting cell percentage in AtpTreg versus other cells of each paired compartment ICI-IA sample in single-cell secreting proteomics. (**F**) Dominant cell clone (defined as cells with > 1% expanded clonotype per sample) percentage in AtpTreg versus other Treg cells of each paired compartment ICI-IA sample in single-cell VDJseq. (**G**) Shared cell clone percentage in AtpTreg cell in single-cell VDJseq of patients with ICI-IA. (**H**) Research flowchart showing grouping of single-cell surface proteomics, single-cell VDJ sequencing, and flow cytometry analysis (FCA), of paired ICI-IA samples of blood as well as synovial fluid (SF) at 4 clinical stages: ICI-naive, preclinical, onset, and 5–7 months follow-up stage after ICI-IA onset. (**I** and **J**) Example gating (**I**) and flow cytometry analysis of AtpTreg cells in 4 patients at 4 clinical stages, gated on Treg cells of peripheral blood. (**K**) Single-cell surface proteomic analysis of AtpTreg cells in 4 patients at 3 clinical stages of paired samples of peripheral blood and SF. (**L**) Clonotype expansion rate of multiple clonotypes found to be persistent between onset and follow up (but were not present at preclinical stage), in paired samples of peripheral blood via single-cell VDJseq in one patient (Patient # 13). *P < 0.05, **P < 0.01, ***P < 0.001, calculated with paired Wilcoxon signed-rank test.

To profile Tregs in multiple compartments (Figure 3A) in patients with ICI-IA (*n* = 4) as contrasting control groups of RA-CA (*n* = 3) and ICI-NC (osteoarthritis in cancer patients with ICI treatment, *n* = 4), we isolated CD4^+^CD25^+^Treg from peripheral blood, tumor, and synovial fluid (SF) samples to study the cross-tissue features of these atypical Treg cells. Tregs underwent Abseq coupled with single-cell VDJ-TCR sequencing (sc-TCRseq), as well as single-cell secreting proteomics. Unbiased clustering of surface proteins autoclustered into 4 clusters visualized as UMAP plot (Supplemental Figure 2, A and B). These clusters were annotated (Figure 3B) as AtpTreg cluster (cluster # 2) and other clusters (# 0, # 1, and # 3) according to the differentially high-expressing proteins (Supplemental Figure 2, B and C).

Overall, in SF, blood, and tumor, the AtpTreg cluster was mainly populated in ICI-IA patient samples as compared with 2 control groups (Figure 3C). Specifically, the AtpTreg cluster was characterized by activated memory effector markers (Figure 3D) consistent with previous findings (e.g., CD40L^+^, ICOS^+^, IL6RA^+^), while other clusters featured high expression of inhibitory markers (Supplemental Figure 2C), including CTLA4, N5TE, NRP1, and LAG3, which are also characteristic of classic Treg cell types. Also, the AtpTreg cluster exhibits similar although minor different expression of markers among the 3 sample sites. The key surface proteins CD137, IL-6R, PD-1, and ICOS were consistently detected across all 3 compartments. Notably, CD11a expression was significantly higher on AtpTregs in SF compared to blood, consistent with an activated, tissue-trafficking phenotype. IL6RA protein was detected in blood and SF but not reliably expressed in tumor-derived AtpTregs, which may reflect compartment-specific biological regulation or technical factors related to enzymatic digestion. Consistent with previous findings, the functional phenotype (Supplemental Figure 2, E–G) was similar among the 3 compartments, harboring high levels of IL-17A-secreting cells (Figure 3E). In single-cell VDJ sequencing of cross-compartment data of patients with ICI-IA, almost all dominant clones (> 1% expanded) were populated in AtpTreg cells of patients with ICI-IA but not in other cell clones (Figure 3F and Supplemental Figure 3, A and B). A comprehensive deep analysis of TCR sequencing data revealed unique molecular signatures in the AtpTreg repertoire. AtpTregs exhibit a distinct nucleotide bias in their CDR3 sequences and dominance of the TRBV as well as TRAV gene combination in clones shared across compartments. The VDJ usage and CDR3 sequence data for dominant clones are provided as Supplemental Data and Supplemental Figure 12. Cross-compartment sharing (defined as identical clonotype in SF, blood, and tumor) of CD137^+^ AtpTreg clonotypes was found in 15.51%–29.90% cells per patient (Figure 3G). These findings suggested homogeneous and expanded AtpTreg cells in the 3 compartments in patients with ICI-IA.

To uncover the relationship between ICI treatment of AtpTreg at the temporal axis and to follow-up the dynamics of AtpTreg cells over the disease trajectory, sequential sampling (Figure 3H) was performed in 8 patients using flow cytometry analysis (*n* = 4) and single-cell TCRseq coupling Abseq (*n* = 4). As compared with ICI-naive or pre-clinical stage, the AtpTreg cell population was significantly increased at disease onset stage with stable high proportion at follow-up stage (Figure 3, I and J). Proportion changes from onset to follow up were validated in another 4 patients via Abseq techniques of SF samples paired with blood samples (Figure 3K). Also, persistent clonotypes were identified in AtpTreg cells from onset to follow-up (Figure 3L and Supplemental Figure 3C), but were not present at the preclinical stage. For instance, one patient exhibited 7 persistent clonotypes at onset and follow-up stage both in blood and in SF samples (Figure 3L). Taken together, these data demonstrate AtpTregs do not arise immediately after ICI administration, but emerge specifically at disease onset.

### AtpTreg is associated with poor arthritis prognosis but better survival outcomes of patients with cancer.

**Figure 4:**
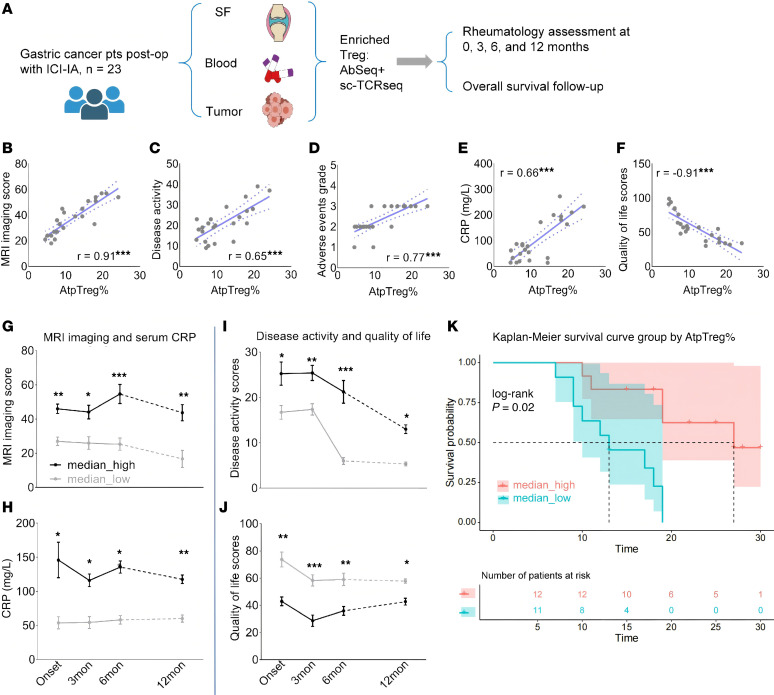
AtpTreg is associated with poor arthritis prognosis but better survival outcomes of cancer patients. (**A**) Research flowchart showing single-cell surface proteomics coupled with Sc-VDJseq of bead-enriched Treg cells from paired samples of blood, synovial fluid (SF), as well as tumor of ICI-IA, serial clinical assessment follow-up protocols, and function experiments, related to Supplemental Table 2. (**B**–**F**) Correlation plot showing the proportion of overall AtpTreg cells in each patient and MRI imaging (WIPE scores, **B**), clinical disease activity (CDAI scores, **C**), adverse event severity (CTCAE scores, **D**), serum CRP values (**E**), and cancer patients’ quality of life (FACT-G scores, **F**). *P < 0.05, **P < 0.01, ***P < 0.001. (**G** and **H**) Dynamics of MRI imaging (WIPE scores, mean ± SEM, **G**) and serum CRP values (mean ± SEM, **H**), of 4 timepoints during 12-month follow up since onset in patients with ICI-IA, categorized by high and low median value of overall AtpTreg cell proportions in each patient. *P < 0.05, **P < 0.01, ***P < 0.001, calculated with independent Wilcoxon signed-rank test. (**I** and **J**) Dynamics of clinical disease activity (CDAI scores, mean ± SEM, **I**) and quality of life (FACT-G scores, mean ± SEM, **J**), of 4 timepoints during 12-month follow-up since onset in patients with ICI-IA, categorized by high and low median value of overall AtpTreg cell proportions in each patient. *P < 0.05, **P < 0.01, ***P < 0.001, calculated with independent Wilcoxon signed-rank test. (**K**) Kaplan-Meier survival curve showing survival time (since ICI treatment) of patients with gastric cancer, categorized by high and low median value of overall AtpTreg cell proportions in each patient, with difference of survival rate calculated with log-rank test (*P* = 0.02).

Given the phenotype as well as clonotype findings of Treg cells of SF, tumor, and blood, we thus interrogated the role of cross-compartment AtpTregs in cancer-related as well as ICI-IA–related outcomes during longitudinal follow-up. We recruited gastric cancer patients with neoadjuvant ICI therapy and sampled their blood, SF, and post-surgical tumor specimens for AtpTreg cell assay (Abseq coupled with scTCRseq, Figure 4A and Supplemental Tables 2–5). We obtained follow‑up information regarding their outcomes (cancer and arthritis) (Figure 4A). Specifically, they were administered rheumatology assessment at 4 timepoints during a 12-month period (onset, 3-month, 6-month, and 12-month since onset), including imaging findings of arthritis, serum CRP levels, clinical arthritis activity (CDAI scores), and adverse event severity (CTCAE grade scores). Considering that the clinical course of ICI-IA usually presents with protracted pathology (16, 17), these parameters comprehensively profile activity dynamics of arthritis at both subjective and objective levels. Articular inflammation was quantified using the OMERACT MRI Whole-Body Score for Inflammation in Peripheral Joints and Entheses (MRI-WIPE), a validated scoring system that assesses and sums four key domains: bone marrow edema (score 0–3), synovitis (score 0–3), soft tissue inflammation at entheses (score 0–3), and entheseal osteitis (score 0–3). In addition, they were followed up for 30 months in terms of overall survival timed from ICI start. A total of 23 patients with gastric cancer were included (10 females, 13 males, mean age 59.35 ± 5.10) treated with neoadjuvant PD-1 or PD-L1 blockade therapy, followed by surgical removal. A reanalysis stratifying patients by specific ICI agent (PD-1/PD-L1 inhibitors) revealed no significant differences in the AtpTreg phenotype, prevalence, or response to therapy across ICI types within our cohort (Supplemental Figures 5–7), supporting the pooling of patients to define a core mechanism. Consistent with our prior conclusion, cross-compartment AtpTreg per patient was found positively and relatively strongly correlated with MRI-imaging score (Figure 4B), disease activity (CDAI) score (Figure 4C), CTCAE grade (Figure 4D), serum CRP levels (Figure 4E),and quality-of-life (FACT-G) score (Figure 4F) at disease onset. When divided by the median percentage of AtpTreg per patient, patients with higher levels of AtpTreg had higher levels of serum CRP (Figure 4G) and MRI (Figure 4H) imaging (WIPE scores) over a 12-month period (objective assessments). Correspondingly, they reported higher levels of disease activity (CDAI scores, Figure 4I) and lower levels of quality of life (FACT-G scores, Figure 4J) over a 12-month period (subjective assessments). Receiver operating characteristic (ROC) curve analysis on Cohort 2 data (Supplemental Figures 7) showed that the cross-compartment AtpTreg frequency is a powerful predictor of poor arthritis activity at 6 months (AUROCs: 0.92 for CDAI, 0.97 for CRP, 0.92 for FACT-G, 0.96 for WIPE) and of good overall survival (AUROC 0.79). Optimal AtpTreg percentage cut-offs were in the range of 8%–11%. These data suggested a prognostic role of AtpTreg for long-course ICI-IA pathology. As for overall survival of cancer patients, it was found that patients with higher levels of cross-compartment AtpTreg frequency had increased overall survival outcomes compared with patients with low levels (Figure 4K). The Kaplan-Meier survival curve demonstrates a statistically significant difference (log-rank test *P* = 0.02). Prior research pointed out that intratumoral Treg may play a key role in diminishing antitumor immunity, but our study suggested that CD137^+^ subset may play opposite roles in patients with ICI-IA. Combined with the finding of persistent existence of AtpTreg cells, these findings collectively suggested AtpTregs as disease signature were associated with poor ICI-IA prognosis but better cancer outcomes in real-world settings. Compartment-specific analysis revealed that AtpTreg frequency in both blood and tumor was significantly associated with improved survival, while SF-derived AtpTreg frequency correlated most strongly with arthritis severity but not survival, suggesting tissue-specific functionality (Supplemental Figures 8–13).

### Anti-IL6R therapy diminished AtpTreg and improved ICI-IA outcomes without compromising cancer survival outcomes.

**Figure 5:**
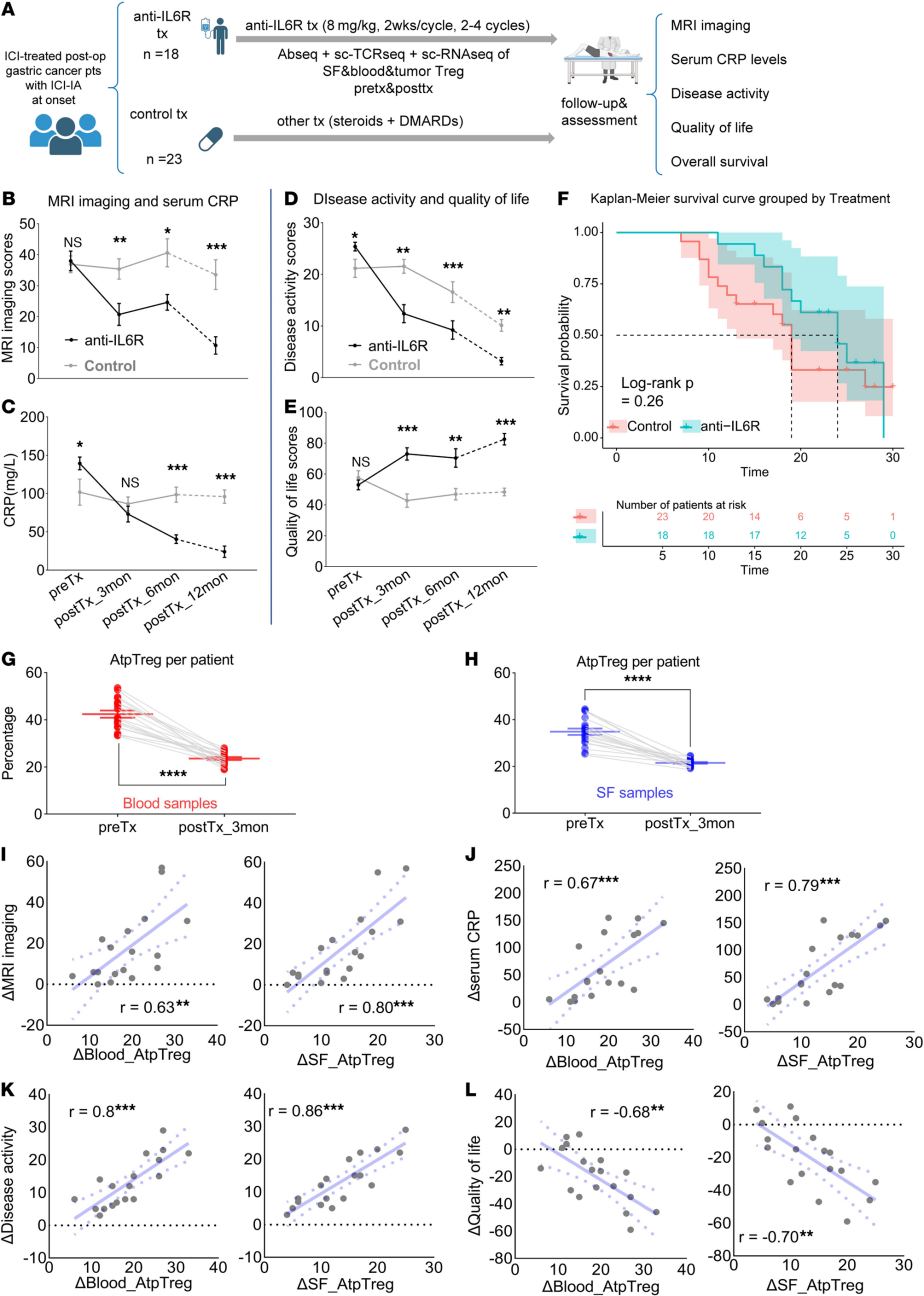
Anti-IL6R therapy diminished AtpTreg cell and improves clinical outcomes. (**A**) Research flowchart of anti-IL6R treatment as compared with standard-practice control group. (**B** and **C**) Dynamics of WIPE scores (**B**) and serum CRP values (**C**) of 4 timepoints during 12-month follow-up since onset in ant-IL6R treatment group and control group. (**D** and **E**) Dynamics of CDAI (**D**) and FACT-G scores (**E**), of 4 timepoints during 12-month follow-up since onset in ant-IL6R treatment group and control group. (**F**) Kaplan-Meier survival curve showing survival time of ant-IL6R treatment group and control group, with difference of survival rate calculated with log-rank test. (**G**) Dynamics of blood-derived AtpTreg over Treg cell proportions from pretreatment to posttreatment samples in these 18 patients treated with anti-IL6R therapy. (**H**) Dynamics of SF-derived AtpTreg over Treg cells proportions from pretreatment to posttreatment samples in these 18 patients treated with anti-IL6R therapy. (**I**) Correlation plot showing the decrease of %AtpTreg cells and WIPE score decrease in each patient from pretreatment to posttreatment follow up. (**J**) Correlation plot showing the decrease of %AtpTreg cells and serum CRP level decrease in each patient from pretreatment to posttreatment follow up. (**K**) Correlation plot showing the decrease of %AtpTreg cells and CDAI score decrease in each patient from pretreatment to posttreatment follow up. (**L**) Correlation plot showing the decrease of %AtpTreg cells and quality of life (FACT-G scores) decrease in each patient from pretreatment to post treatment follow-up. *P < 0.05, **P < 0.01, ***P < 0.001, ****P < 0.0001, calculated with independent Wilcoxon signed-rank test.

ICI-IA has substantially restricted its use and has brought significant burdens during cancer management. Observational reports as well as open-labeled trials showed potential benefits of anti-IL6R treatment (tocilizumab), yet its mechanism and long-term effects remained elusive (18, 19). The use of tocilizumab for managing ICI-IA, while not a globally standard oncology protocol, was a physician-guided clinical decision for patients significantly impacted by ICI-IA. All patients provided explicit informed consent for this specific off-label therapeutic intervention (see Informed Consent Form in Supplemental Materials). Given the general role of Treg cells in inflammatory pathology and considering our unique finding that AtpTreg cells may serve as a pathogenic role with stable IL6R expression, we enrolled an independent cohort (cohort 3) of patients with ICI-IA with gastric cancers treated with tocilizumab at disease onset (Figure 5A and Supplemental Figure 4B) and studied Treg cell dynamics before and after treatment. A total of 18 tocilizumab-treated patients were included with new-onset ICI-IA after checkpoint blockade therapy (see demographics in Supplemental Table 3). Overall, compared with standard antiinflammatory management, the anti-IL6R therapy group had significantly more improved clinical outcomes of ICI-IA during a 12-month period of follow up after disease onset, manifested objectively with more decreased level of CRP and MRI imaging scores (objective assessments) at 3-month, 6-month, and 12-month timepoints (Figure 5, B and C). Clinical assessment indicated corresponding higher improvement, as suggested by disease activity (CDAI) scores and quality-of-life (FACT-G) scores (subjective assessments; Figure 5, D and E). Surprisingly, there is no significant difference in overall survival outcomes between standard treatment group and the anti-IL6R therapy group (Figure 5F). These findings suggested that anti-IL6R therapy is a superior therapy against ICI-IA in patients with cancer without significantly affecting oncology-related outcomes of cancer patients. For these 18 patients, single-cell proteomics (Abseq) were performed on isolated Treg cells in SF as well as blood samples before and after tocilizumab treatment. We found that AtpTreg cell proportions decreased both in SF (Figure 5G) and blood samples (Figure 5H), which was in line with our previous finding that AtpTreg proved as a disease signature. Such a level of decrease (Δ) is correlated with changes in Δ imaging scores, Δ CDAI scores, serum Δ CRP, and Δ quality of life in these 18 patients (Figure 5, I–L). Combined with previous findings, these data suggested that AtpTreg cells, which express IL6R, may be a therapeutic target by tocilizumab without affecting cancer-related outcomes.

**Figure 6:**
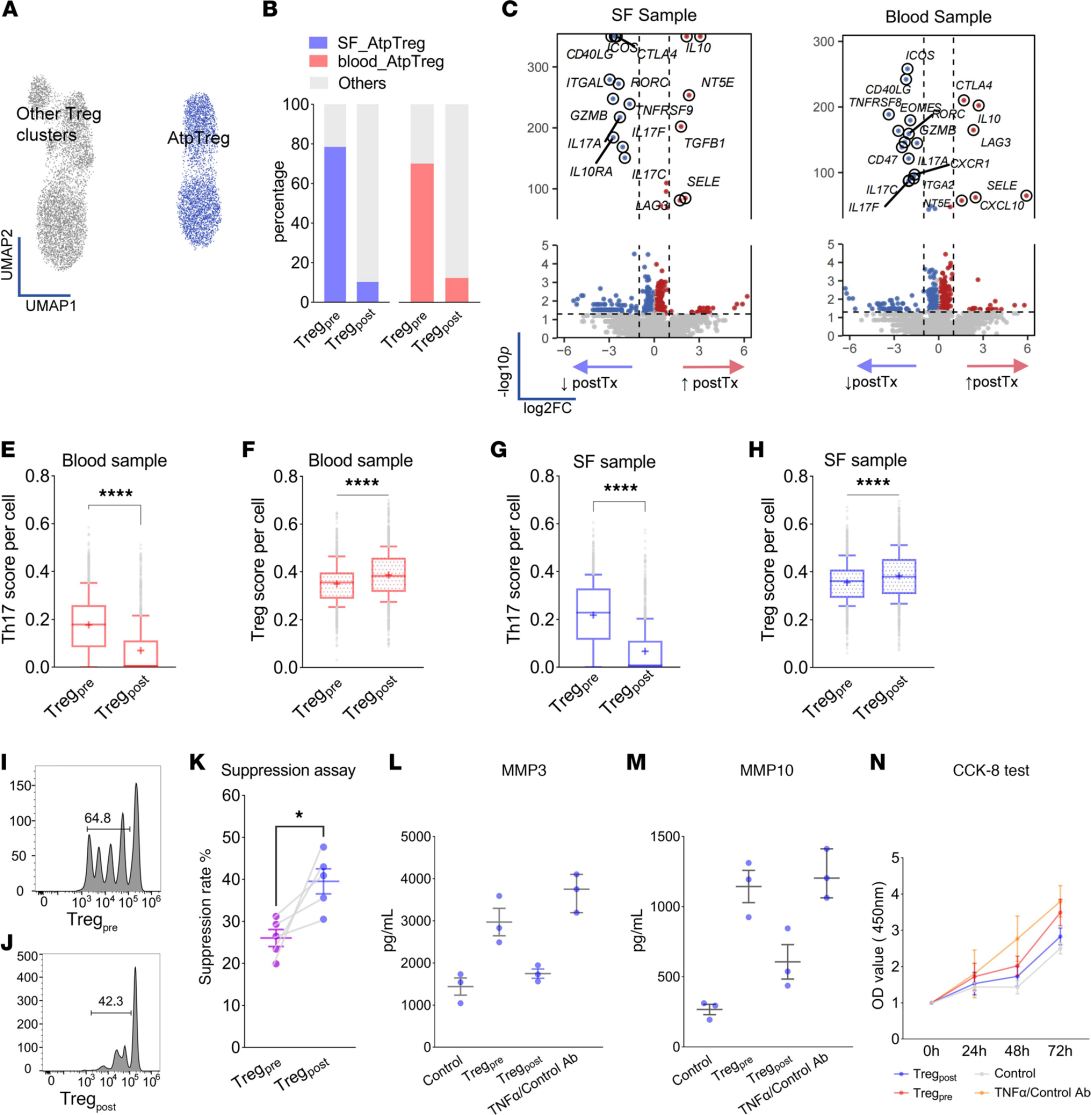
Changes of genes and function in Treg cells before and after anti-IL6R treatment. (**A**) UMAP clustering defined by single-cell RNAseq of bead-enriched Treg cells of blood+SF samples of ICI-IA before and after anti-IL6R treatment (*n* = 3). (**B**) Distribution of AtpTreg cell cluster across treatment conditions (Tregpre and Tregpost) in blood and SF sample Tregs. (**C** and **D**) Differentially expressed genes across treatment conditions (Tregpre and Tregpost) in blood (**C**) and SF (**D**) samples. (**E**–**H**) Canonical Treg/Th17 Signature scores by clusters in blood (**E** and **F**) and in SF (**G** and **H**) Tregpre and Tregpost. ****P < 0.0001, calculated using paired Wilcoxon test. (**I**–**K**) Treg suppression assay with Tregs for Tresp from healthy controls. CFSE labelled T responder (Tresp) cells were cocultured with Tregs isolated from pretreatment and posttreatment blood samples. FlowJo figures are shown for CFSE negative cells at a Tresp‑to‑Treg ratio of 1:4 (**I** and **J**) and suppression analysis plot shown (**K**). (**L** and **M**) ELISA test of MMP-3 (**L**) and MMP-10 (**M**) in MH7A in coculture system, grouped by blood Tregpre and Tregpost, as compared with controls. (**N**) CCK-8 test of cell counts of MH7A in each group of coculture system, grouped by Tregpre and Tregpost in blood, as compared with controls. *P < 0.05, calculated using paired Wilcoxon test.

Single-cell RNA-seq were performed in 3 patients before and after tocilizumab in SF and blood samples to illustrate the genes changed before and after therapy. Overall, treatment caused drastic clustering of Tregs on the UMAP plot (Figure 6A). AtpTregs were decreased both in SF and in blood samples after treatment (Figure 6B), which is in line with prior findings on the proteomic level. Other genes that were changed included *GZMB*, *IL17A*, *CD40LG*, *EOMES*, *RORC*, *IL10*, and *CTLA4* (Figure 6, C and D). These genes were annotated as activation markers, Th17-related pathway, and autoimmune pathway (Figure 6, E and F). Also, Th17 scores decreased while Treg scores increased after treatment in both blood and SF samples (Figure 6, G–J). We then tested how Treg cell function changes after anti–IL-6R treatment. Tregs were isolated from ICI-IA patients either before (Treg_pre_) or after (Treg_post_) anti-IL-6R treatment, and cocultured with Tresp of healthy donors. Treg_post_ showed increased ability to inhibit Tresp proliferation compared with Treg_pre_ (Figure 6, K–M). Furthermore, Treg_post_ failed to increase proliferation of synovial cell lines or increase expression of metalloproteinases (MMP3/10) expression (Figure 6, L–N). In an in vitro assay (Supplemental Figure 13), treatment of purified CD137-conventional Tregs from healthy donors with IL-6 alone was sufficient to induce upregulation of CD137, demonstrating that a core feature of the AtpTreg phenotype can be induced by a key cytokine present in ICI-IA inflammation (Supplemental Figure 13). These data suggested that anti-IL6R treatment restored Treg phenotypes and functions and effectively decreased intraarticular inflammation.

## Discussion

Our study provides unprecedented insights into the pathogenesis of immune checkpoint inhibitor–induced inflammatory arthritis by identifying a novel proinflammatory CD137^+^AtpTreg subset, which exhibits a pronounced Treg/Th17 overlap phenotype. The work integrates single-cell multi-omics across paired peripheral blood, synovial fluid, and tumor tissues — a design that captures systemic immune dynamics inaccessible to conventional approaches. Biologically, we resolve a key paradox in ICI toxicology: while these therapies typically deplete Tregs, we demonstrate clonally expanded, tissue-trafficking CD137^+^ AtpTregs specifically enriched in patients with ICI-IA. These cells exhibit a unique phenotype (e.g., *IL6R*^+^*CTLA4*^–^*IL17A*^+^, with high *RORC* gene expression) and functional switch from immunosuppression to synovial inflammation promotion, thus illustrating Treg plasticity in irAEs. Clinically, we establish this subset’s dual prognostic role as its frequency correlates with worse arthritis severity but better cancer survival, thus linking irAE pathology to antitumor immunity. Crucially, our translational validation in an independent cohort proves that anti-IL6R therapy selectively ablates AtpTregs, concurrently improving articular outcomes and cancer-related quality of life without compromising antitumor immunity. Collectively, these findings position AtpTregs as both a mechanistic cornerstone and actionable target in ICI-IA.

The hybrid proinflammatory-regulatory phenotype of CD137^+^ AtpTregs in ICI-IA parallels emerging evidence that Treg plasticity is dynamically regulated by epigenetic and cytokine cues. Recent studies demonstrate that inflammatory milieus (e.g., IL-6/STAT3 signaling) can destabilize Tregs by erasing FOXP3 methylation, driving Th17-like reprogramming (20), while tumor microenvironments may license alternative plasticity pathways such as activation of effector genes (10, 21). Our findings extend these paradigms by revealing that ICI therapy itself triggers a unique Treg diversion — clonally expanded CD137^+^ AtpTregs coexpress FOXP3 with IL-17A/ROR-γt yet lack classical suppression markers (CTLA4/LAG3), suggesting a therapy-induced epigenetic reset distinct from chronic inflammation or tumor-driven adaptation. We did not detect appreciable expression of the IL23R gene in our scRNA-seq data, suggesting the canonical IL-23/IL-23R pathway may not be dominant in this context. Our preliminary data suggest that the functional impairment of AtpTregs may extend to cytokine suppression, reminiscent of the disassociation between suppression of proliferation and cytokine secretion observed in Tregs from patients with active rheumatoid arthritis (22–24). Notably, the persistence of these cells after ICI cessation hints at stable transcriptional reprogramming. Quantitative PCR analysis on sorted AtpTregs showed no difference in exon 2 transcript levels of FOXP3 compared with other Tregs. Their IL-6R dependence mirrors the IL-6 axis implicated in RA-associated Th17-like Tregs (15, 20), but with compartmentalized pathogenicity (synovial-tropic clones) and paradoxical survival benefits (systemic tumor control). This duality underscores the need for context-specific modulation — targeting plasticity drivers (e.g., IL-6R) without broad Treg depletion — to balance irAE management and anticancer immunity. While AtpTregs share a Th17-like phenotype with Tregs described in autoimmunity and cancer (25), they are distinguished by their ICI-induced origin, CD137^+^ surface signature, and systemic clonal expansion across tumor and synovium (26–28). Most notably, their frequency paradoxically links severe arthritis with improved cancer survival, revealing a unique symbiosis between irAE pathology and antitumor immunity in the ICI setting. We acknowledge that anti–IL-6R therapy exerts pleiotropic effects; our data identify modulation of AtpTregs as a possible mechanism associated with efficacy in ICI-IA.

The paradox of clonal CD137^+^ AtpTregs — driving inflammatory arthritis yet correlating with superior cancer survival — reveals a fundamental symbiosis between irAE pathology and antitumor immunity in ICI therapy (8). Our data demonstrate that clonally expanded CD137^+^ AtpTregs (15.5%–29.9% cross-tissue clonal sharing) systemically infiltrate tumors, blood, and synovium, linking joint inflammation to broader immune activation. These clones emerge after ICI and persist alongside active arthritis (5–7 months) with a positive association with prolonged survival, suggesting their systemic expansion inadvertently sustains anti-tumor responses while locally provoking toxicity. The observation that anti–IL-6R therapy improves arthritis without compromising survival, despite depleting circulating and synovial AtpTregs, suggests that the tumor microenvironment may retain sufficient antitumor immunity, or that reducing systemic inflammation improves overall immune fitness. This symbiosis was evidenced by therapeutic validation in tocilizumab intervention, proving that targeted disruption of the IL-6 axis can possibly uncouple irAE toxicity from antitumor efficacy. Interestingly, IL-6 signaling has been shown to correlate to cytotoxic storm (29, 30). Importantly, unlike unselective immunosuppression-like steroids, other Treg subtypes (like CTLA4^+^ inhibitory Tregs) were unaffected. Thus, pathway-specific modulation, rather than global immunosuppression such as steroids, represents a viable strategy to balance irAE management and cancer control in ICI recipients.

While our study establishes AtpTregs as key mediators of ICI-induced arthritis, several limitations warrant consideration. First, the exclusive focus on PD-1/PD-L1 inhibitor–treated cohorts precludes exploration of other immunotherapy effects (e.g., CTLA-4 blockade), which may distinctively modulate Treg dynamics (11). Second, generalizability is constrained by the predominant enrollment of patients with gastric cancer during clinical validation or anti-IL6R therapy to control for confounding bias. Validation in other cancer types with diverse ICI regimens is needed, although the incidence of ICI-IA in our cohorts was comparable to internationally reported data (31–33). Third, although we demonstrate multicompartmental clonal expansion, the developmental origin of AtpTreg clones remains unresolved—whether they arise from thymic-derived Tregs, peripherally converted Tregs, or tumor-infiltrating precursors requires lineage-tracing studies. Fourth, the antigen specificity driving clonal expansion is uncharacterized, leaving open whether shared tumor/self-antigens initiate crossreactivity. Fifth, while we show impaired suppressive function of AtpTregs from blood and synovial fluid, functional comparison with tumor-derived AtpTregs was not feasible due to limited longitudinal sampling before and after surgery. Future studies are needed to address this. Finally, while tocilizumab efficacy was shown in a real-world cohort, comparative trials against alternative biologics (e.g., IL-17 inhibitors) with larger sample size are warranted to define optimal therapy and proper cancer management modality.

In conclusion, this work establishes proinflammatory AtpTregs and IL-6R signaling as central drivers of checkpoint inhibitor–induced arthritis, providing a mechanistic roadmap for targeted immunomodulation to manage this debilitating side effect while preserving anti-tumor immunity.

## Methods

### Sex as a biological variable.

This study included both male and female participants across all 3 cohorts (Supplemental Tables 1–3). Sex was recorded as part of the patient demographic data but was not analyzed as a biological variable in the primary immunological or survival analyses due to the limited sample size for sex-stratified statistical comparisons. The fundamental immune pathways investigated in this study — specifically, the identification of atypical Tregs and their response to IL-6R blockade — are not known to be sex specific. Therefore, the findings are expected to be mechanistically relevant for both sexes.

### Human participants and settings.

We conducted a multicenter cohort study to explore the dynamics of systemic immune cells in patients with inflammatory arthritis following checkpoint blockade treatment of solid cancer patients. The study involved 3 independent cohorts with serial sampling of multiple-tissue/system immune cells combined with clinical assessment. Cohort 1 included patients with multiple cancer types from the Affiliated Cancer Hospital of Shantou University Medical College, enrolled between June 2021 and May 2022. Cohort 2 comprised gastric cancer patients from Sun Yat-Sen Cancer Center and Cancer Hospital of Shantou University Medical College, recruited from January 2022 to April 2023. Cohort 3 consisted of patients at Cancer Hospital of Shantou University Medical College, First Affiliated Hospital of Zhengzhou University and Hainan Hospital of PLA General Hospital enrolled between November 2022 and July 2024 (Supplemental Tables 1–3 and 7).

Patients with ICI-IA after ICI treating malignancy were diagnosed by experienced rheumatologists and oncologists, with all clinical signs evaluated according to Common Terminology Criteria for Adverse Events (CTCAE, version 5.0). The date of ICI-IA diagnosis was established by referencing outpatient and inpatient medical records. Two cancer control groups were included in cohort 1. Seropositive RA patients co-morbid with cancers fulfilled the 2010 ACR/EULAR classification criteria. ICI-NC were patients who were cancer patients on ICI therapy but without an irAE. In Cohort 3 we recruited ICI-IA patients receiving anti-IL6R therapy and followed their immune profiles before and after treatment. To assess overall survival differences between treated patients and controls, we only included gastric cancer patients. Key exclusion criteria from this study were (a) arthroscopy site infection; (b) cancer metastasis to arthroscopy knee joint; (c) co-morbid other autoimmune musculoskeletal pathology that obscures immune profile analysis; (d) failed follow-up assessment in longitudinal studies.

### Sampling procedure of tumor, blood, and synovial fluid.

Peripheral blood samples were obtained from participants via venipuncture. Synovial fluid samples were obtained from participants via arthrocentesis. Before arthrocentesis (Supplemental Tables 4–6), paired peripheral blood samples were collected from the patients with active ICI-IA, active RA, or ICI-NC patients. Fresh tumor samples at the post-ICI stage were collected as surgical specimens from the operation surgery departments (34). Specimens were minced and incubated in RPMI-1640 medium (Gibco, #11875093), including 10% heat-inactivated fetal bovine serum (FBS) (Gibco, #10099141), Normocin (10 μg/ml) (InvivoGen, #ant-nr-1), DNAse I (2 μg/ml) (Roche, #4716728001), and collagenase IV (1 mg/ml) (Worthington Biochem, #LS004186). The mixture was shaken at 37°C for 30 minutes.

Cell suspension was then filtered through a 70-μm filter and washed with RPMI-1640 media. Cells were then resuspended in a 37.5% Percoll density gradient medium (Cytiva, #17-0891-01) and centrifuged (690 *g*, 25 minutes, room temperature) to obtain lymphocytes. These were purified with Ficoll-Paque plus gradient (Cytiva, #17144003) and LeucoSep tubes according to the manufacturer’s manual (Greiner Bio-One, #227290). Cells were then washed in flow cytometry buffer (PBS with 2% FBS and 2 mM EDTA) at 4°C and blocked with human Fc Block (1:100, flow cytometry buffer; Miltenyi Biotec, #130-059-901). Viable cells were purified from dead cells with Ficoll-Paque Plus medium (GE Healthcare), followed by storage in liquid nitrogen for batch analyses. Treg cells were enriched by positive selection with CD4^+^ CD25^+^CD127dimRegulatory T Cell Isolation Kit II supplemented with REAlease CD25, and CD137 beads were added when necessary.

### Clinical assessment.

Disease activity was quantified using the Clinical Disease Activity Index (CDAI, score range 0–76), a validated composite measure calculated as the sum of four components (35): tender joint count (0–28 joints), swollen joint count (0–28 joints), self-reported overall assessment (0–10 visual analog scale), and physician-reported overall assessment (0–10 visual analog scale). CDAI evaluations were performed at ICI-IA and RA-CA patient and every clinical visit thereafter during follow-up. Serum C-reactive protein (CRP) levels were measured using standardized high-sensitivity assays (lower detection limit 0.03 mg/L), with values ≥ 5 mg/L considered clinically relevant for systemic inflammation.

Health-related quality of life was assessed using the Functional Assessment of Cancer Therapy-General (FACT-G) questionnaire (36). The FACT-G assesses 5 domains: Physical Well-Being, Social and Family Well-Being, Relationship with Doctor, Emotional Well-Being, and Functional Well-Being, with a 27-item scale (score range 0–108), with higher scores indicating better quality of life.

Tumor response and survival outcomes were tracked through routine oncology visits, during which time follow-up was carried out on a one-month basis for 3 months since neo-adjuvant ICI therapy, and on a 3-month basis since surgery until 36 months after surgery. Overall survival (OS) was calculated from cancer diagnosis to death from any cause, with final censoring confirmed alive date for patients lost to follow-up. All analyses adhered to intention-to-treat principles. Longitudinal specimen collection (peripheral blood, synovial fluid, tumor tissue), if any, was synchronized with clinical assessments.

### Magnetic resonance imaging scoring.

Using OMERACT (Outcome Measures in Rheumatoid Arthritis Clinical Trials) MRI-WIPE (MRI Whole-body score for Inflammation in Peripheral joints and Entheses), ICI-IA is assessed in the bone from the articular surface/entheseal insertion to 1 cm depth (37, 38).

Bone marrow edema, scoring 0–3, depends upon oedematous bone volume, compared to the assessed bone volume on all images: normal (0 point), mild (1 point, ~1/3 of bone), moderate (2 points, 1/3 to 2/3 of bone), severe (3 points, > 2/3 of bone).

Soft tissue inflammation is defined as inflammation inside ligaments and surroundings to 1 cm from entheseal insertion (0 to 3 points): normal (0 point), mild (1 point), moderate (2 points), severe (3 points), reflecting the volumetric extent of inflammation relative to maximum potential involvement.

Synovitis was quantified throughout synovial compartments, with grading determined by the volume of inflamed tissue: No synovial hyperplasia (0 point), progressive involvement by thirds of maximum potential volume (1 to 3 points).

For bilateral knee examinations, the protocol evaluated synovitis at 2 joint sites, osteitis at 10 sites, entheseal inflammation at 10 sites, entheseal osteitis at 10 sites, giving a max total score of 96.

For synovitis or tissue inflammation, evaluation was on post-contrast T1-weighted sequences. Osteitis was assessed via STIR/T2-fat-saturated (T2FS) sequences. When contrast-enhanced imaging was unavailable, STIR/T2FS sequences were utilized for all inflammatory domains per OMERACT recommendations.

### Single-cell VDJ repertoire, surface proteomic, and RNA sequencing.

Single-cell libraries were constructed via the BD Rhapsody Express system (BD Biosciences, #633707) according to the manufacturer protocol (BD Biosciences). Cells were labeled with cell-specific tag and donor-specific identification tag (BD Biosciences, #633781). Cells were then put onto stain buffer wash (BD Biosciences, #554656), followed by pooled incubation of Fc block and per-cell surface protein antibody labeling (BD AbSeq Ab-Oligos master mix). Cells were pooled to reach 1000–10,000 counts in 600–700 mL buffer fluid. Primed cartridges were applied to load pooled cells (incubated at room temperature) and then to load cell beads after washing. These were incubated and washed twice with sample buffer, and then cells were lysed. Reverse transcription was carried out after retrieval of beads and washing (Supplemental Table 8).

Subsequent steps were related to VDJ and surface proteomic sequencing library protocol to generate libraries using the BD Rhapsody system. During reverse transcription, Poly-T Template Switching Oligo was added to allow for finding VDJ recombination events. Nucleotides were denatured, hybridized, and went through Klenow extension (New England Biolabs, #M0212L) and treatment with Exonuclease. cDNAs were prepared in nested PCR reaction, followed by index PCR with targeted mRNA, Amplification Kit (BD Rhapsody, #633774), and Immune Response Panel (BD Rhapsody, #633750). Library concentration was calculated with dsDNA Assay Kit (Qubit, ThermoFisher, #Q32851). Quality control was performed with TapeStation with High Sensitivity D5000 ScreenTape (Agilent 2200).

Sample tags, mRNA, and antibody barcode reads were trimmed to 75 nt, and VDJ libraries trimmed to 225 nt, followed by sequencing on a Novaseq SP flow at 75*225. Refined calling was disabled on the analysis pipeline (BD Rhapsody, https://www.sevenbridges.com/bdgenomics/).

### Single-cell secreting proteomics.

Cells were obtained by centrifugation at 4°C, 1900 *g* for 10 minutes. Upon experiment, cells were loaded into 96 wells pre-coated with anti-CD3 (OKT3, ThermoFisher) and anti-CD28 (ThermoFisher) at the density of 1 × 105/200 μl, and cultured in completed RPMI media (Fisher Scientific) plus 10 ng/mL IL-2 (Biolegend) at 37°C, 5% CO2 for 16 hours. Cells were then loaded onto an IsoCode Chip and incubated at 37°C, 5% CO_2_ for 16 hours. Following this incubation, secreted proteins from 100–2000 cells/sample were captured by the 32-plex antibody barcoded chip and analyzed by backend fluorescence ELISA-based assay.

### Single-cell data analysis.

Preprocessing and cluster analysis was performed with the “Seurat” package in R Statistics. Cells without any features and features with < 10 cells were excluded from matrix. Dimension reduction was first performed by principal component analysis (PCA) and then by Uniform Manifold Approximation and Projection (UMAP) embeddings to see unsupervised clustering of single cells. PCA was carried out with all features for analysis. Elbow plot was adopted to identify useful components, and 4 components were applied for UMAP plot in each analysis, with resolution set as 0.1. Default-parameter “FindAllMarkers” function was adopted to mark specific cluster with 25% difference from all other clusters. For secreting and surfacing proteomics, global scaling was performed prior to log-transformation and centering from counts of surface barcode sequencing or illuminant secreting index. Secondary filtering was performed on surface proteomics to remove mitochondrial and ribosomal expression-related cells. Cluster 2 was annotated as AtpTreg based exclusively on its distinct gene expression profile from scRNA-seq data. The KEGG pathway enrichment analysis was performed using the list of up-regulated differentially expressed genes (DEGs) in Cluster 2 compared to all other Treg clusters (C0, C1, C3, C4). DEGs were identified using the FindAllMarkers function in Seurat with thresholds: min.pct > 0.1, log_2_ Fold-Change > 0.1, and adjusted *P*-value < 0.05 (Bonferroni correction).

Single-cell VDJ files went through the default-parameter Cellranger VDJ 4.0 pipeline to generate clonotype information. Reads were referred to and annotated by human reference in the IGBLAST database of NCBI. Information was then manually entered into single-cell proteomic Seurat object as combined phenotype-clonotype matching in each cell with the help of cell barcodes. Clonotype was defined as combined information of VDJ sequences shared by all such cells. Cells with identical clonotypes were defined as the same cell clone. These data allowed for association analysis between VDJ clonotypes and cell phenotype (coupled surface proteomics). Cells with both chains of VDJ sequences passed quality control for analysis. As such, clonotypes were defined as paired VDJ sequences of both chains in TCRfαβ, TCRγδ, or BCR-heavy/light chains. Clonal expansion was defined as the frequency of cells with one same clonotype and cells with one same CDR3 clonotype identical clonotypes were defined as the same cell clone. Dominant clonotypes were defined as those with > 1% expanded per patient sample. Persistent clonotypes were defined as identical clonotypes shared between sampling timepoint at follow-up and onset samples.

### Treg suppression assay.

The PAN T Cell Isolation Kit Human (Miltenyi Biotec, cat. 130-096-535) was used to obtain the untouched T cell fraction (responder cells, Tresp) from 4 healthy controls [mean age 41.3 (range 22–54) years], according to instructions from the manufacturer (Miltenyi Biotec). Tresp cells (1 × 10^6^ cells/ml) were rested overnight in TexMACS (Miltenyi Biotec, cat. 130-097-196) medium containing 5% human AB serum and 50 U/ml rIL2 (Miltenyi Biotec) at 37°C and 5% CO_2_. These cells were then stained with the CFSE Cell Proliferation Kit according to instructions from the manufacturer (Biolegend, cat. 423801). Tresp and Tregs were dissolved to a concentration of 5 × 10^5^ cells/ml in TexMACS medium supplemented with 50 U/ml rIL2, 5% human AB serum, and 1% penicillin–streptomycin (Treg suppression medium). For the suppression assay, cells were activated with 3 μl/ml Immunocult Human CD3/CD28 T cell Activator (Stemcell Technologies, cat. 10971) and co-cultured in Tresp-to-Treg ratios of 1:1, 4:1, and 8:1 for 5 days at 37°C and 5% CO_2_ (Supplemental Figures 14–16).

### Cellular experiments.

Proliferative capacity in each group was assessed by the CCK-8 method at 4 time points: 0 hours, 24 hours, 48 hours. After TNF-α stimulation, MH7A cells (Procell system, #CL-0747, RRID: CVCL_0427) were added with 10 μL/well CCK-8 solution (Beyotime, #C0038) for 3 hours. The microplate reader was utilized for determination of absorbance at 450 nm. MH7A cells without TNF-α stimulation were set as negative control. Proteins were measured using the Proximity Extension Assay (PEA, Olink Explore-3072 panel) according to the manufacturer’s instructions. We also measured concentrations of metalloproteinases (MMP-10, MMP-3) using ELISA kits (#ab100602 of Abcam, and Wuhan Boster) according to the manufacturer’s instructions. The magnetic bead enrichment strategy involved a 3-step process: (a) Depletion of non-CD4^+^ and CD127^+^ cells using the Regulatory T Cell Isolation Kit II (Miltenyi catalog 130-094-775); (b) Positive selection of CD25^+^ cells using the REAlease (Miltenyi catlog 130-133-522) CD25 MicroBead Kit to obtain label-free Tregs; (c) Isolation of CD137^+^ AtpTregs from label-free Tregs using CD137 microbeads (Miltenyi catlog 130-093-476). The purity of the bead-enriched CD137^+^ Treg population, assessed by flow cytometry, was approximately 66.35% within the live cell gate. Cell viability was assessed immediately after isolation using Trypan Blue exclusion. For single-cell experiments, dead cells and debris were removed using Ficoll-Paque Plus density gradient centrifugation, and only live cells were processed for library construction.

### Flow cytometry staining.

Cells were stained with the following antibodies (30–40 minutes, 4°C) indicated for surface protein staining: anti-CD3-APC Cy7 (SK7, BioLegend), anti-CD4-BV510 (SK3, BioLegend), anti-CD137-BV421 (BD), antiCD127- Spark.NIR.685 (G043H7, BioLegend), anti-CD25-BB515 (HI100, BD), anti-FOXP3-PE (BD, RRID: AB_1645508). Cells were then washed and acquired by a spectral flow cytometer (Cytek). The data were analyzed by FlowJo 10.4.2. Directly conjugated murine IgG1 and IgG2 were adopted as background stain. For fluorescence-activated cell sorting, we used a BD FACSAria II flow cytometer configured with 4 lasers. The purity of the resulting CD137^+^ Treg population, assessed by flow cytometry, was approximately 98%~99% within the live cell gate. For other flow cytometry analyses, we used a Cytek Aurora spectral flow cytometer configured with five lasers. Intact cells were gated via forward scatter and side scatter (FSC-A/SSC-A). Doublets were dropped via FSC-H/FSC-W followed with SSC-H/SSC-W gates. In cellular experiments, cells were sorted into cold RPMI with 10% FBS. No materials were received as gifts from individual researchers.

### Statistics.

Continuous variables were presented with mean ± SEM. Categorical variables were presented with counts and percentage. The difference of percentages was compared with McNemar nonparametric test. Continuous variables were compared with Wilcoxon signed-rank tests to test distributions. For paired comparisons, paired Wilcoxon signed-rank tests were performed to compare cross-compartment or time-dependent changes. Batch effects arising from different patients and sample processing batches were removed using the Harmony algorithm applied to the principal components derived from the integrated Seurat object. Data visualization was done using the OmicStudio tools (39) or Graphpad Prism 9.5.1. Fold change was adopted to describe the extent of difference, and all *P* values in multiple comparison statistics were moderated into FDR (t)-*P* values by the Benjamini-Hochberg procedure. A *P* value less than 0.05 was considered significant. For patient-level analysis, we did not calculate the patient sample sizes needed to reach statistical significance to prioritize sequential sampling of the low-incidence pathology. No randomization or blinding happened in participant enrollment, and we did not recruit patients from known randomized trials.

### Study approval.

This study involving human participants was reviewed and approved by the Institutional Review Board of the Affiliated Cancer Hospital of Shantou University Medical College (Shantou, Guangdong, China; Approval No. ST-ZLY-2020-716) Reporting adhered to the Strengthening the Reporting of Observational Studies in Epidemiology (STROBE) checklist for cohort studies. All procedures were performed in accordance with the ethical standards of the Declaration of Helsinki. Written informed consent was obtained from all individual participants prior to their inclusion in the study. This study is registered at ClinicalTrials.gov (NCT07357636).

### Data availability.

Raw and processed Sequencing data is publicly available at the GSA-Human (Genome Sequence Archive for Human) at China’s National Genomics Data Center under accession number HRA017294. The proteomics data is available in the PRoteomics IDEntifications Database (PRIDE) under accession number PAD000037. Sharing of raw RNA sequencing data was not allowed by the informed consent signed by patients enrolled in this study. Values for all data points in graphs and reported means are provided in the Supporting Data Values file. Any additional information required to reanalyze the data reported in this paper is available from the lead contact upon request.

## Author contributions

AZ, RL, S Weng, and FD conceptualized and designed the study, and reviewed and revised the manuscript; Yifei Ma, DZ, YJ, JZ, YX, XL, ZG, WJ, LW, QH and BC designed the data collection instruments, carried out the initial data collection and processing. NL, S He, Yue Ma, TW, YW, YSN, NF, S Wang, Jinjian Li, GZ and HL carried out analyses and wrote the first draft of the manuscript. Yan Li, J Lv, GJ, PZ, J Lin, Jin Li, Yiming Li, CL, MY, S Huang and J Lu coordinated and supervised data collection and critically reviewed the manuscript for important intellectual content. All authors approved the final manuscript as submitted and agreed to be accountable for all aspects of the work. The work reported in the paper has been performed by the authors unless specified in the text.

## Conflict of interest

The authors declare no competing interests.

## Funding support

- The National Natural Science Foundation of China General Fund Project (32370962 to RL).
- National Natural Science Foundation of China Youth Science Fund Project (82102687 to Yifei Ma, 82201922 to AZ).
- Regional joint key support project of National Natural Science Foundation of China (U23A20428 to RL).
- Natural Science Foundation of Fujian Province (2025J01753 to YM).
- Joint Funds for the Innovation of Science and Technology, Fujian Province (2023Y9054 to Y.M, 2024Y9220 to SW).

## Supplementary Material

Supplemental data

ICMJE disclosure forms

Supporting data values

## Version 1 05/12/2026

In-Press Preview

## Version 2 07/01/2026

Electronic publication
