# Supplementary material for: IL-6 receptor blockade impedes proinflammatory atypical Treg subset associated with immune checkpoint inhibitor–induced inflammatory arthritis
Source: J Clin Invest. 2026 May 12;136(13):e200135. doi: 10.1172/JCI200135 (PMC13318110; doi:10.1172/JCI200135)
Supplement: Supplemental data [file jci-136-200135-s007.pdf]

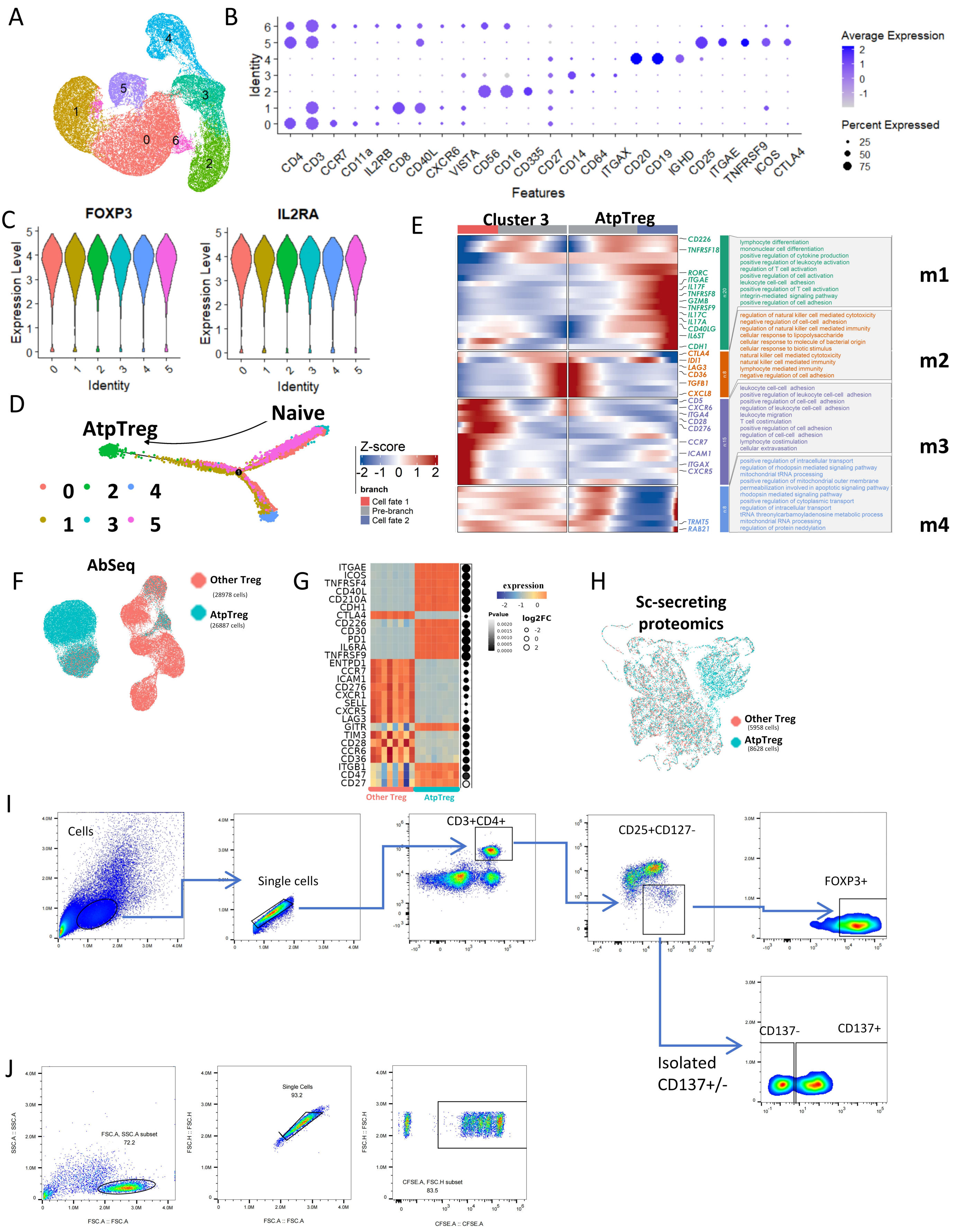

A
B
Average Expression
Percent Expressed
Identity
Features
CD4 CD3 CCR7 CD11a IL2RB CD8 CD40L CXCR6 VISTA CD56 CD16 CD335 CD27 CD14 CD64 ITGAX CD20 CD19 IGHD CD25 ITGAE TNFRSF9 ICOS CTLA4
C
FOXP3
IL2RA
Expression Level
Identity
D
AtpTreg
Naive
Z-score
branch
Cell fate 1
Pre-branch
Cell fate 2
E
Cluster 3
AtpTreg
m1
m2
m3
m4
F
AbSeq
Other Treg
(28978 cells)
AtpTreg
(26887 cells)
G
expression
Pvalue
log2FC
Other Treg
AtpTreg
H
Sc-secreting proteomics
Other Treg
(5958 cells)
AtpTreg
(8628 cells)
I
Cells
Single cells
CD3+CD4+
CD25+CD127-
FOXP3+
CD137-
CD137+
Isolated CD137+/-
J
FSC.A, SSC.A subset
72.2
Single Cells
93.2
CFSE.A, FSC.H subset
83.5

Supplementary Figure 1

A, UMAP clustering defined by single-cell surface proteomics of peripheral blood mononuclear cell samples of ICI-IA, RA-CA&ICI-NC patients, related to Figure 1B-D.

B, Signature proteins of each cluster were depicted as the bubble plot, related to Figure 1B-D.

C, Violin plot showing expression level of FOXP3 and IL2RA in each sc-RNAseq-defined cluster.

D-E, Pseudotime analysis reveals a T cell activation-related role of AtpTreg cells. Cluster 1 represents the AtpTreg cell population of interest, while Cluster 3 comprises naïve Treg cells characterized by CCR7 expression. Heatmap of differentially expressed genes (DEGs; $q < 0.05$) between two pseudotime branches, highlighting four gene modules (M1–M4) with distinct expression dynamics along the trajectories toward two cell fates (Cell fate 1 and Cell fate 2). M1 corresponds to the differentiation path toward AtpTreg cells, showing significant enrichment in pathways related to T cell activation, cell adhesion, and immune effector functions.

F, UMAP clustering defined by single-cell surface proteomics of bead-enriched AtpTreg (red) and other Treg (green) cells, showing AtpTreg and other Treg sample groups.

G, Heatmap of average expression of each Abseq markers per sample.

H, UMAP clustering defined by single-cell secreting proteomics of bead-enriched AtpTreg (red) and other Treg (green) cells, showing AtpTreg and other Treg sample groups.

I, Gating strategy of Treg cells in peripheral blood samples of ICI-IA patients in Flow cytometry to show FOXP3 expression level and CD137+/- subset sorting strategy in suppression assay, related to Figure1O-P.

J, Gating strategy of CFSE cells in peripheral blood samples of ICI-IA patients in Flow cytometry, related to Figure1O-P.

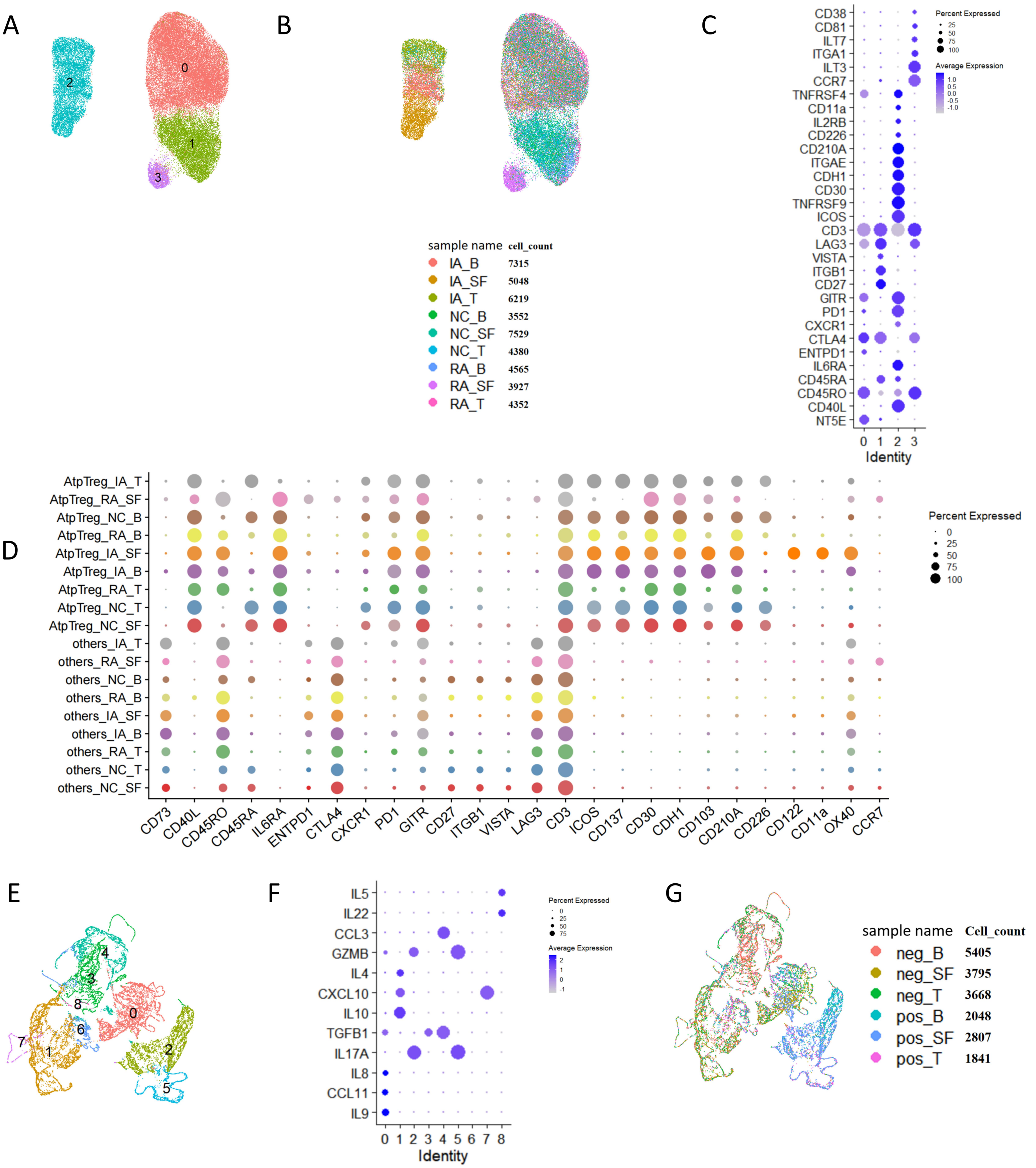
A
0
1
2
3
B
sample name cell_count
IA_B 7315
IA_SF 5048
IA_T 6219
NC_B 3552
NC_SF 7529
NC_T 4380
RA_B 4565
RA_SF 3927
RA_T 4352
C
CD38
CD81
ILT7
ITGA1
ILT3
CCR7
TNFRSF4
CD11a
IL2RB
CD226
CD210A
ITGAE
CDH1
CD30
TNFRSF9
ICOS
CD3
LAG3
VISTA
ITGB1
CD27
GITR
PD1
CXCR1
CTLA4
ENTPD1
IL6RA
CD45RA
CD45RO
CD40L
NT5E
0 1 2 3
Identity
Percent Expressed
25
50
75
100
Average Expression
1.0
0.5
0.0
-0.5
-1.0
D
AtpTreg_IA_T
AtpTreg_RA_SF
AtpTreg_NC_B
AtpTreg_RA_B
AtpTreg_IA_SF
AtpTreg_IA_B
AtpTreg_RA_T
AtpTreg_NC_T
AtpTreg_NC_SF
others_IA_T
others_RA_SF
others_NC_B
others_RA_B
others_IA_SF
others_IA_B
others_RA_T
others_NC_T
others_NC_SF
CD73
CD40L
CD45RO
CD45RA
IL6RA
ENTPD1
CTLA4
CXCR1
PD1
GITR
CD27
ITGB1
VISTA
LAG3
CD3
ICOS
CD137
CD30
CDH1
CD103
CD210A
CD226
CD122
CD11a
OX40
CCR7
Percent Expressed
0
25
50
75
100
E
0
1
2
3
4
5
6
7
8
F
IL5
IL22
CCL3
GZMB
IL4
CXCL10
IL10
TGFB1
IL17A
IL8
CCL11
IL9
0 1 2 3 4 5 6 7 8
Identity
Percent Expressed
0
25
50
75
Average Expression
2
1
0
-1
G
sample name Cell_count
neg_B 5405
neg_SF 3795
neg_T 3668
pos_B 2048
pos_SF 2807
pos_T 1841

Supplementary Figure 2

A, UMAP clustering defined by single-cell surface proteomics of bead-enriched Treg cells from paired samples of blood, synovial fluid (SF), and tumor of ICI-IA, RA-CA, and ICI-NC, related to Figure 2B.

B, Cell count of each group and sample analyzed in single-cell surface proteomics of bead-enriched Treg cells, related to Figure 2B.

C, Signature proteins of each cluster were depicted as the bubble plot, related to Figure 2B.

D, Signature proteins of other Treg clusters in blood, SF, and tumor samples of ICI-IA patients, depicted as the bubble plot, related to Figure 2D.

E, UMAP clustering defined by single-cell secreting proteomics of bead-enriched AtpTreg and other Treg cells in paired samples of blood, tumor, and SF, showing cell clusters, related to Figure 2E.

F, Signature proteins of each cluster of single-cell secreting proteomics were depicted as the bubble plot, related to Figure 2E.

G, Cell count of each group and sample analyzed in single-cell secreting proteomics of bead-enriched AtpTreg and other Treg cells, related to Figure 2E.

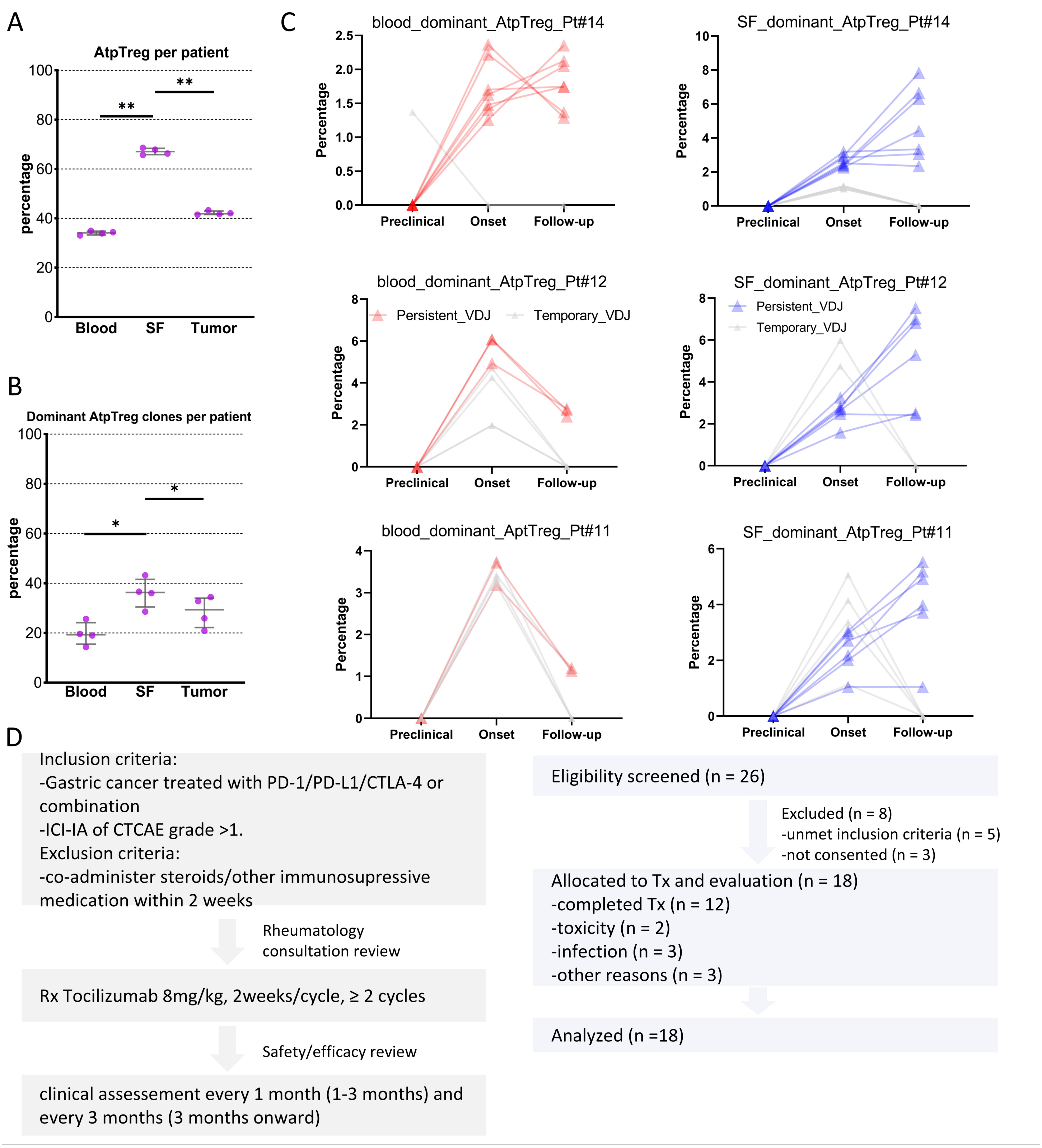
A
AtpTreg per patient
percentage
Blood
SF
Tumor
B
Dominant AtpTreg clones per patient
percentage
Blood
SF
Tumor
C
blood_dominant_AtpTreg_Pt#14
SF_dominant_AtpTreg_Pt#14
blood_dominant_AtpTreg_Pt#12
SF_dominant_AtpTreg_Pt#12
blood_dominant_AptTreg_Pt#11
SF_dominant_AtpTreg_Pt#11
Percentage
Preclinical
Onset
Follow-up
Persistent_VDJ
Temporary_VDJ
D
Inclusion criteria:
-Gastric cancer treated with PD-1/PD-L1/CTLA-4 or combination
-ICI-IA of CTCAE grade >1.
Exclusion criteria:
-co-administer steroids/other immunosupressive medication within 2 weeks
Rheumatology consultation review
Rx Tocilizumab 8mg/kg, 2weeks/cycle, ≥ 2 cycles
Safety/efficacy review
clinical assessement every 1 month (1-3 months) and every 3 months (3 months onward)
Eligibility screened (n = 26)
Excluded (n = 8)
-unmet inclusion criteria (n = 5)
-not consented (n = 3)
Allocated to Tx and evaluation (n = 18)
-completed Tx (n = 12)
-toxicity (n = 2)
-infection (n = 3)
-other reasons (n = 3)
Analyzed (n =18)

Supplementary Figure 3

A, AtpTreg cell per patient of ICI-IA group in each compartment sample, showing that SF has high AtpTreg population, related to Figure 2C. *$p < 0.05$, **$p < 0.01$, ***$p < 0.001$, calculated with independent Wicoxon signed-rank test; ns, not significant.

B, Dominant AtpTreg cell per patient of ICI-IA group in each compartment sample, showing that SF has high dominant AtpTreg population, related to Figure 2F. *$p < 0.05$, **$p < 0.01$, ***$p < 0.001$, calculated with independent Wicoxon signed-rank test; ns, not significant.

C, Clonotype expansion rate of multiple clonotypes from preclinical stage to follow-up, in paired samples of peripheral blood via single-cell VDJseq in another 3 patients (Pt#11, 12, and 14), related to Figure 2L.

D.Treatment Recruitment strategies of ICI-IA patients receiving anti-IL6R treatment

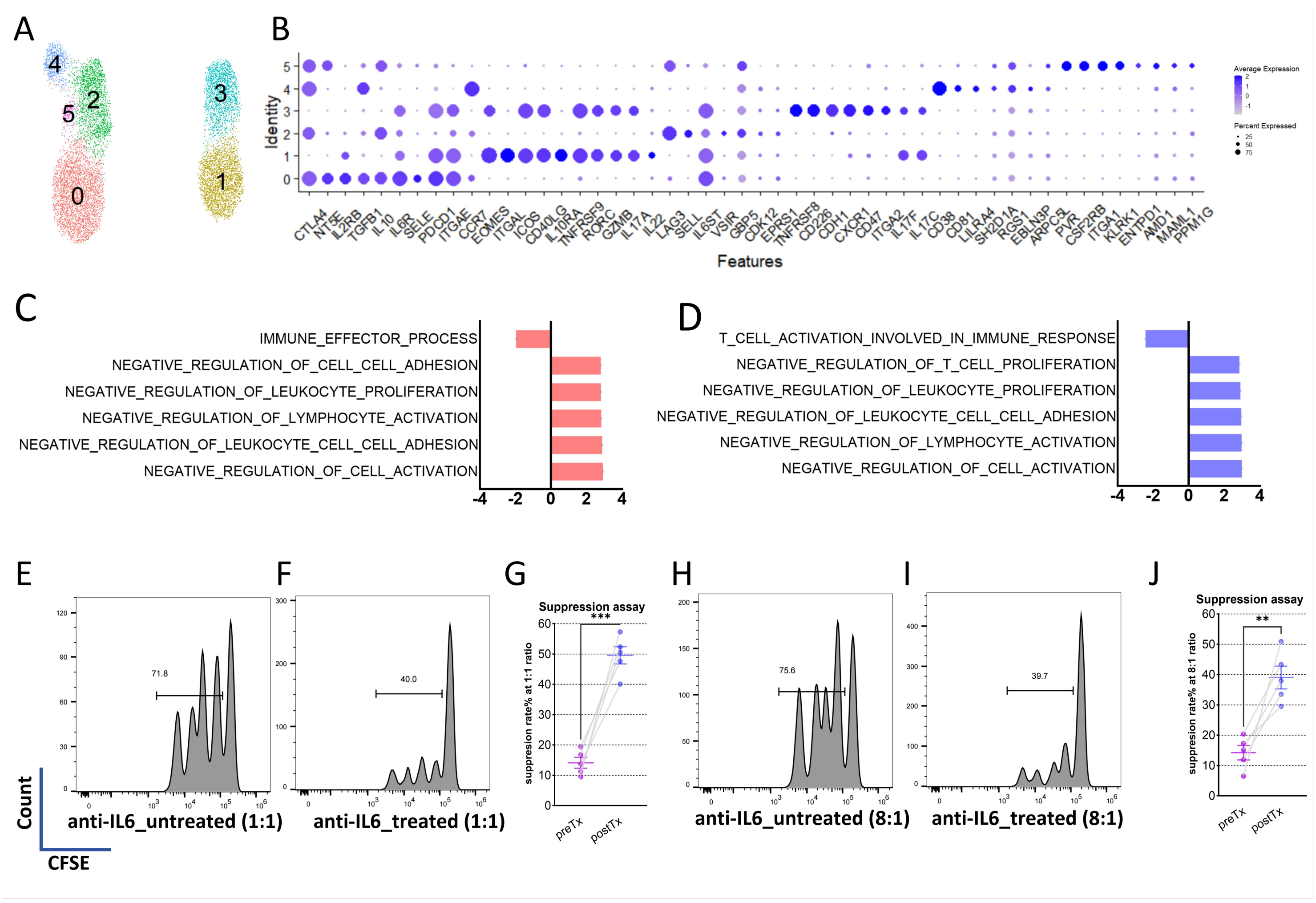

Supplementary Figure 4

A, UMAP clustering defined by single-cell RNAseq of bead-enriched Treg cells of blood+SF samples of ICI-IA before and after anti-IL6R treatment (n = 3), related to Figure 5A and B.

B, Signature proteins of each cluster were depicted as the bubble plot.

C-D, Gene set enrichment analysis (GSEA) of differentially expressed genes (DEGs) across treatment conditions (PreTx vs. PostTx) in blood (C) and synovial fluid (D) highlights predominant enrichment of IL-17–related pathways.

E-J, Treg suppression assay with Tregs for Tresp from healthy controls. CFSE labelled T responder (Tresp) cells were co-cultured with Tregs from Tregs isolated from pre-treatment and posttreatment blood samples. FlowJo figures are shown for CFSE negative cells in 1:1 (E-F) and 1:8 ratio (H-I) and suppression analysis plot shown (G,H). **p < 0.01, ***p < 0.001, calculated using paired Wilcoxon test.

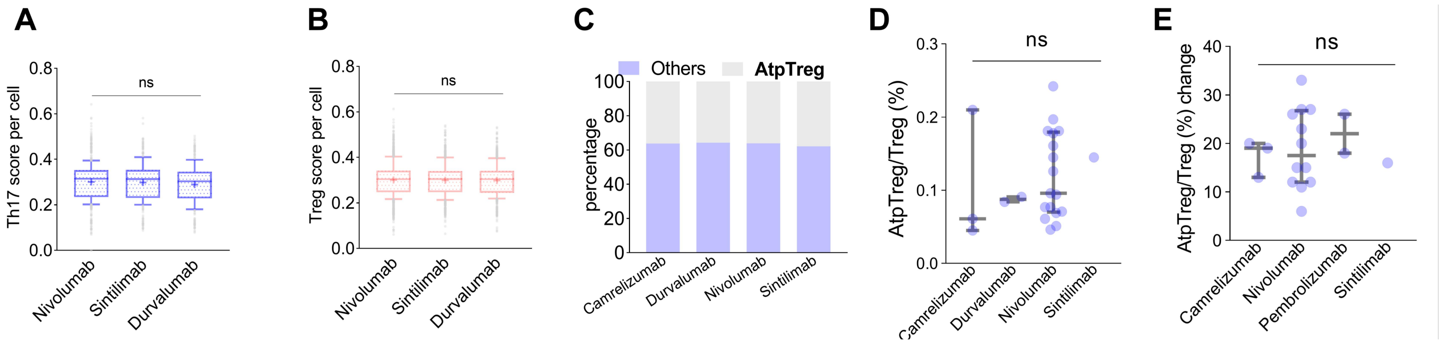

Supplementary Figure 5. Analysis of AtpTreg Phenotype and Prevalence Stratified by ICI Type

(A, B) Single-cell transcriptomic analysis of Cohort 1 (bead-enriched Tregs). Box plots show the distribution of (A) Treg signature scores and (B) Th17 signature scores for cells within the AtpTreg cluster, stratified by the ICI administered to the patient. No significant differences were found (Kruskal-Wallis test).

(C, D) Analysis of cross-compartment AtpTregs in Cohort 2. (C) Stacked bar chart showing the proportion of patients with detectable cross-compartment AtpTregs, grouped by ICI (Chi-square test, p = ns). (D) Scatter plot showing the percentage of AtpTregs per patient, grouped by ICI. Horizontal bars indicate the median. No significant differences were found (Kruskal-Wallis test).

(E) Analysis of therapeutic response in Cohort 3. Scatter plot shows the change in AtpTreg percentage in synovial fluid before and after anti-IL-6R therapy, grouped by the initial ICI responsible for inducing ICI-IA. Horizontal bars indicate the median. The reduction in AtpTregs was consistent across groups, with no significant difference (Kruskal-Wallis test).

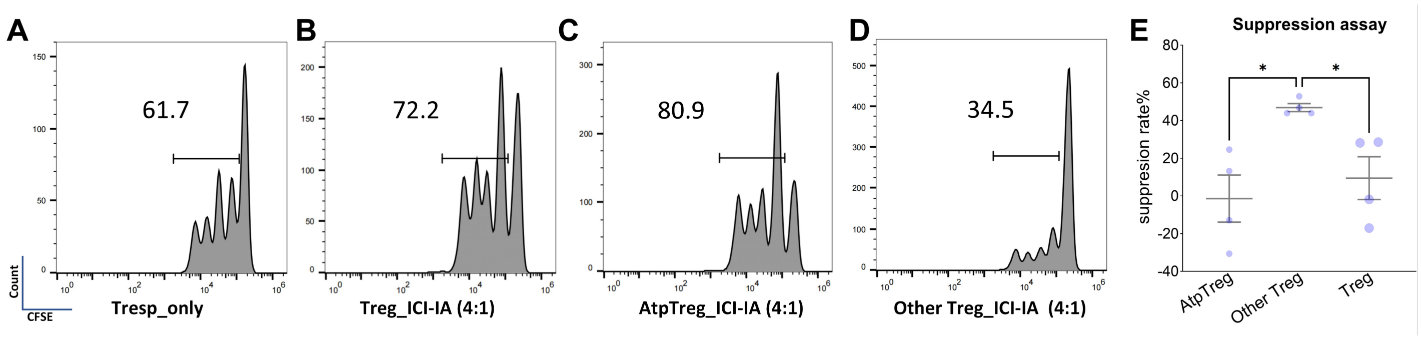

Supplementary Figure 6. Functional Suppression Assay of AtpTregs from Synovial Fluid

(A-D) Representative flow cytometry histograms of CFSE dilution demonstrating the proliferative capacity of healthy donor responder T cells (Tresp) under different co-culture conditions at a 4:1 (Tresp:Treg) ratio: (A) Tresp only; (B) Co-culture with total Tregs from ICI-IA patients; (C) Co-culture with AtpTregs from ICI-IA patients; (D) Co-culture with Other Tregs from ICI-IA patients.

(E) Quantitative analysis of Treg suppression capacity. Scatter plot (n = 4 independent experiments using cells from 4 patients) shows the suppression rate of Tresp proliferation by different Treg subsets: AtpTregs, total Tregs, and Other Tregs from ICI-IA patients. Data are presented as mean ± SEM; *$p < 0.05$ indicates statistically significant differences between groups as determined by non-parametric test with Bonferroni correction.

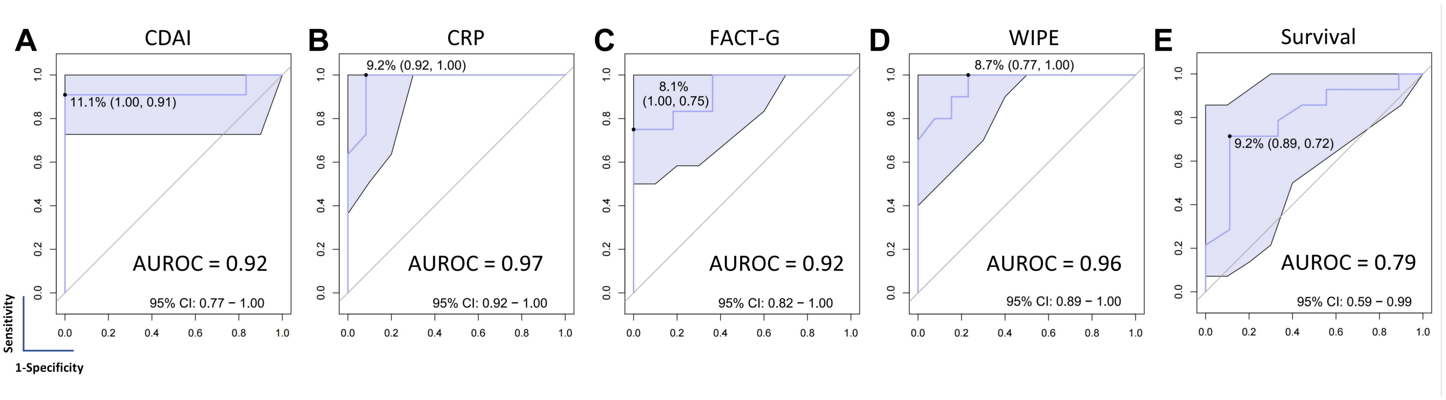

Supplementary Figure 7. Cross-compartment AtpTreg Frequency Predicts Both Arthritis and Survival Outcomes

A-D. Prediction of poor inflammatory arthritis outcome at 6 months, defined as a value worse than the cohort median for (A) Clinical Disease Activity Index (CDAI), (B) serum C-reactive protein (CRP), (C) quality of life (FACT-G), and (D) MRI inflammation (WIPE score).

E. Prediction of good overall survival, defined as survival longer than the cohort median of 19 months. For each ROC curve, the area under the curve (AUROC), the optimal AtpTreg% cut-off value (below reference line), and its corresponding sensitivity and specificity are indicated (above reference line). The grey line represents reference line.

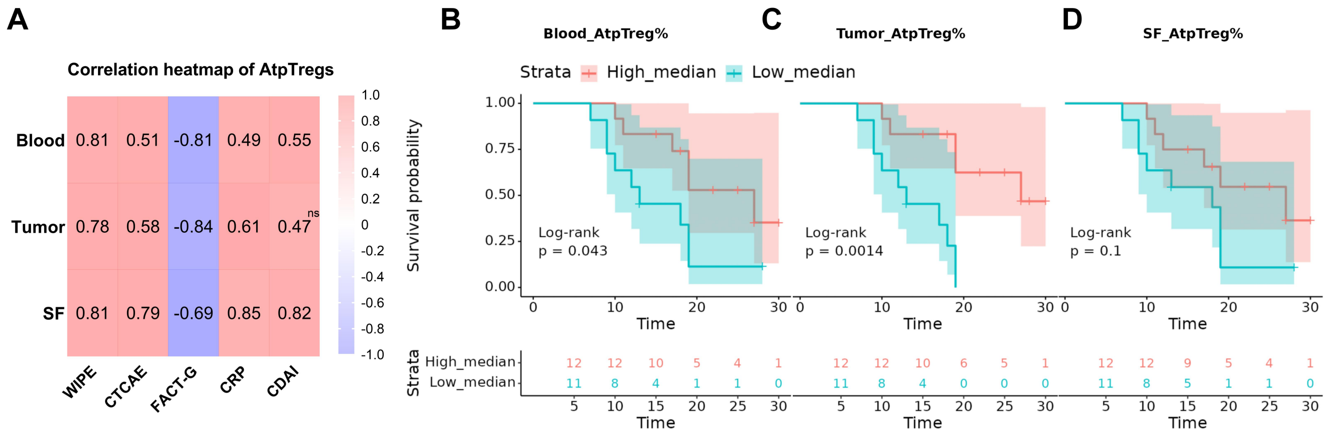

Supplementary Figure 8. Compartment-Specific Analysis of AtpTreg Association with Arthritis and Survival

(A) Spearman correlation heatmap of AtpTreg frequency from blood, tumor, and synovial fluid (SF) with clinical parameters of inflammatory arthritis (WIPE, CTCAE, FACT-G, CRP, CDAI) measured at disease onset. Red indicates positive correlation, blue indicates negative correlation.

(B-D) Kaplan-Meier survival curves for patients stratified by high vs. low AtpTreg frequency based on cohort median from different compartments: (B) peripheral blood, Log-rank p=0.043, (C) tumor, Log-rank p=0.0014, and (D) synovial fluid, Log-rank p=0.1. The number of patients at risk is shown below each graph.

A

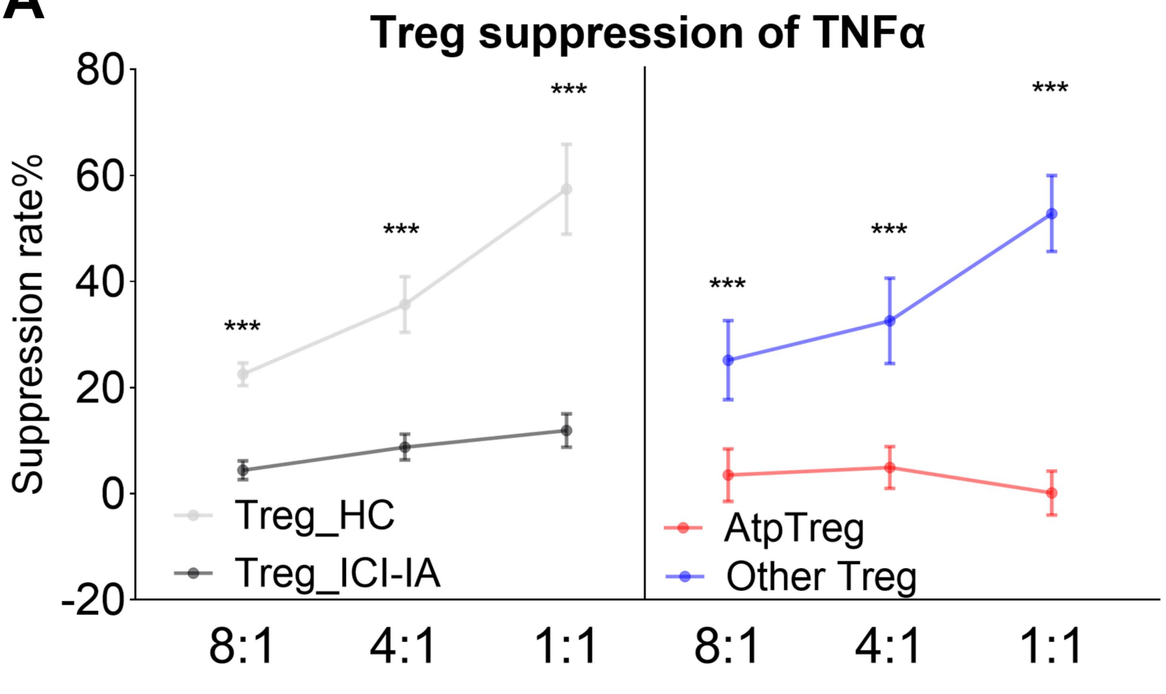

Supplementary Figure 9. Assessment of Treg-mediated suppression of TNF-α secretion.

(A) Dose-response curves depicting the percentage of TNF-α suppression across the three co-culture ratios for each Treg group. Suppression percentage was calculated relative to the TNF-α level from Tresp cells alone.

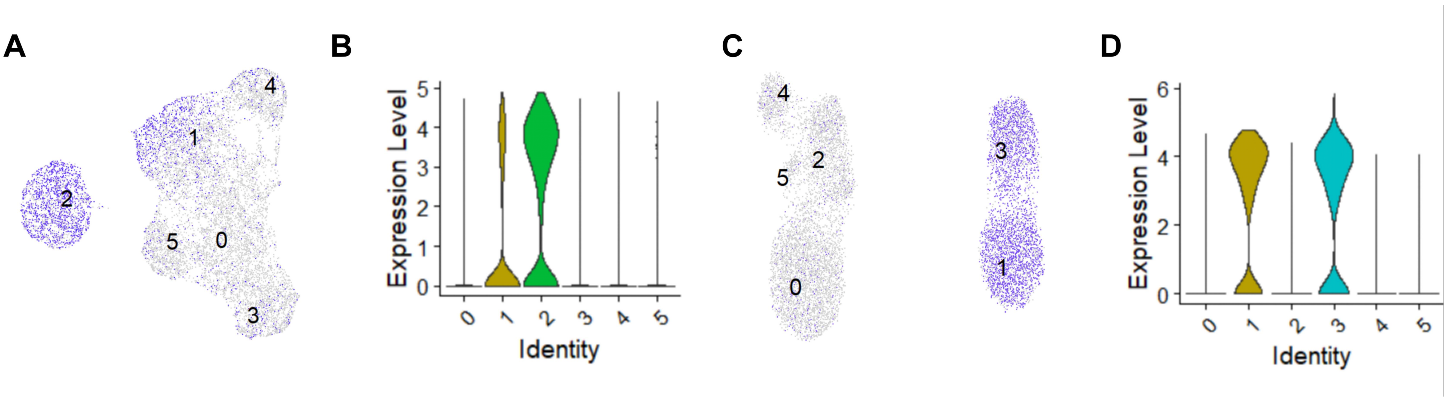

Supplementary Figure 10. RORC Expression Defines the AtpTreg Population and is Modulated by Anti-IL-6R Therapy

(A-B) Analysis of the discovery cohort (Cohort 1). (A) UMAP projection of bead-enriched Tregs from peripheral blood, colored by RORC expression level. The AtpTreg cluster (Cluster #2) shows specific and high expression of RORC. (B) Violin plot quantifying the expression level of RORC across the annotated Treg clusters, confirming significantly higher expression in Cluster #2 (AtpTreg).

(C-D) Analysis of the treatment cohort (Cohort 3). (C) UMAP projection of Tregs from blood and synovial fluid samples, colored by RORC expression. (D) Violin plot quantifying the expression level of RORC in cells.

A

Primer	Sequence (5'-3')	Product
FOXP3_exon2-F1	CTGCACCCAAAGCCTCAGAC	114 bp
FOXP3_exon2-R1	GTGGCATGGGGTTCAAGGA	
hGAPDH-F1	TTCGTCATGGGTGTGAACCA	170 bp
hGAPDH-R1	GTCTTCTGGGTGGCAGTGAT	
hACTB-F1	AGACCTGTACGCCAACACAG	89 bp
hACTB-R1	CCAGGGCAGTGATCTCCTTC	

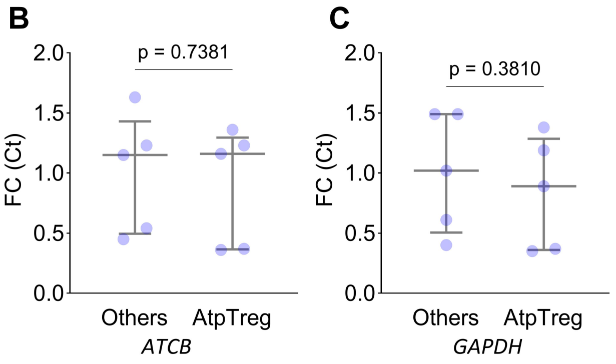

Supplementary Figure 11. Expression of Exon 2-Containing FOXP3 Transcript in Sorted AtpTregs

Analysis of FOXP3 exon 2 expression in fluorescence-activated cell sorted (FACS) AtpTregs (CD137^{+}) versus other Tregs (CD137^{-}) from ICI-IA patient PBMCs (n = 5).

(A) Schematic of the qPCR primer design. The forward (F1) and reverse (R1) primers specifically amplify the region spanning exon 2 of the human FOXP3 gene.

(B-C) Relative expression of FOXP3 exon 2 normalized to (B) ACTB (β-actin) and (C) GAPDH. Each dot represents a biological replicate from an individual patient. Horizontal bars indicate the median. No statistically significant difference was observed between the two groups (Wilcoxon signed-rank test, $p > 0.05$).

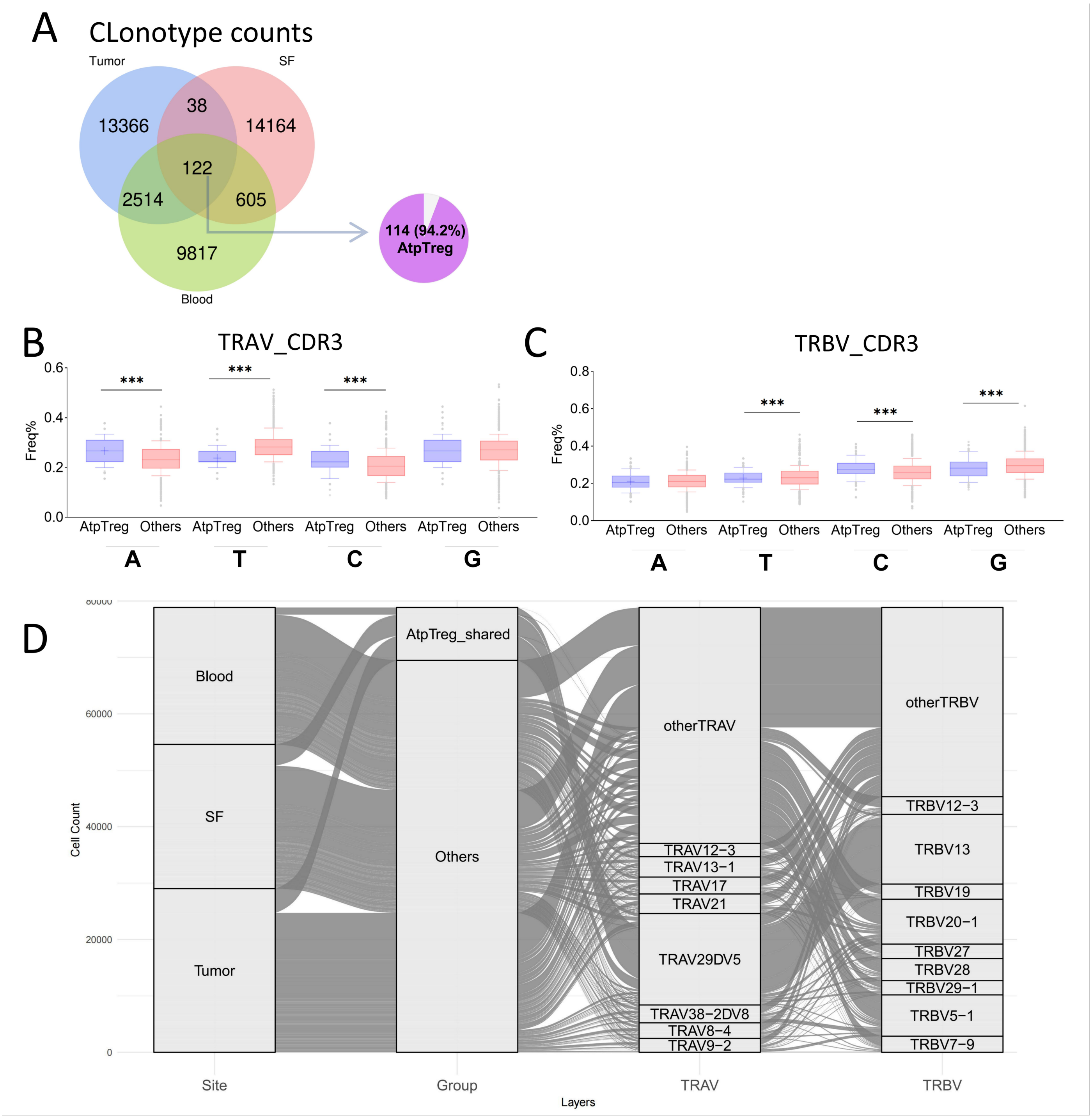

A
CLonotype counts
Tumor
SF
Blood
13366
38
14164
122
2514
605
9817
114 (94.2%)
AtpTreg
B
TRAV_CDR3
Freq%
AtpTreg Others AtpTreg Others AtpTreg Others AtpTreg Others
A T C G
C
TRBV_CDR3
D
Cell Count
Site Group TRAV TRBV
Layers
Blood
SF
Tumor
AtpTreg_shared
Others
otherTRAV
TRAV12−3
TRAV13−1
TRAV17
TRAV21
TRAV29DV5
TRAV38−2DV8
TRAV8−4
TRAV9−2
otherTRBV
TRBV12−3
TRBV13
TRBV19
TRBV20−1
TRBV27
TRBV28
TRBV29−1
TRBV5−1
TRBV7−9

Supplementary Figure 12. In-depth Analysis of TCR Repertoire in AtpTregs Reveals Distinct Clonal and Molecular Features

(A) Venn diagram showing the distribution of clonotypes across three compartments (peripheral blood, synovial fluid, and tumor). The overlapping regions represent shared clonotypes identified in multiple compartments. The proportion and counts of AtpTreg clonotypes among the shared clonotypes is indicated as purple.

(B-C) Nucleotide composition analysis of CDR3 sequences at single-cell resolution. (B) Frequency of A, T, C, G bases in α-chain CDR3 sequences comparing shared AtpTreg clones versus other T cells. (C) Frequency of A, T, C, G bases in β-chain CDR3 sequences. Data are presented as box (median) and 10%-90% whiskers; *$p < 0.01$, t-test with FDR correction.

(D) Sankey plot illustrating TCR V gene usage patterns in shared clones. The flow depicts the relationship from tissue site (left) to cell group (AtpTreg vs. others) to TRAV gene usage to TRBV gene usage. The plot highlights the predominant convergence of AtpTregs onto specific V gene combinations, particularly the TRBV13_TRAV29DV5 pairing.

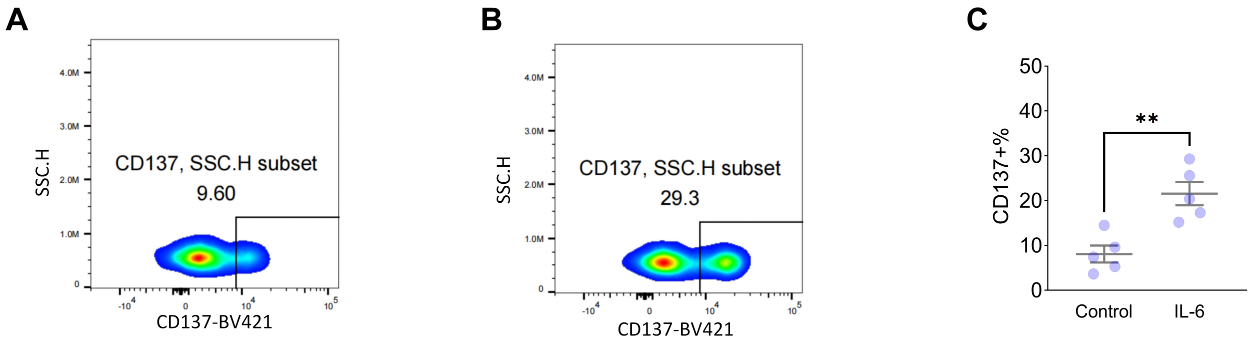

Supplementary Figure 13. In vitro CD137 induction on CD137- Tregs by IL-6 stimulation.
(A-B) Representative flow cytometry plots showing CD137 expression on gated FOXP3+ Tregs. Purified CD137+ Tregs from healthy donors were cultured for 48 hours under different conditions: (A) Unstimulated control media; (B) Stimulated with IL-6 (50 ng/mL). Plots are pre-gated on live, single cells.
(C) Quantification of CD137+ Treg induction. Bar graph shows the mean percentage (± SEM) of CD137+ cells within the Treg population under each condition from 5 independent donor experiments. Statistical significance was determined with non-parametric test. P values are indicated.

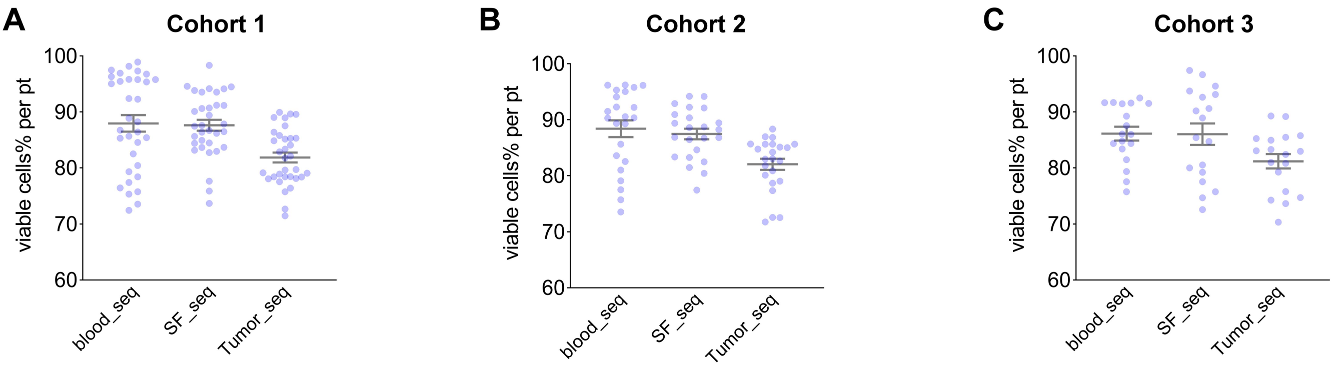

Supplementary Figure 14. Viability of isolated cells from multiple compartments.

(A-C) Cell viability of each cohort patient sample was assessed using Trypan Blue exclusion and counted on a hemocytometer. Each data point represents the viability from an individual patient sample. The horizontal lines represent the mean ± standard error of the mean (SEM) for each group.

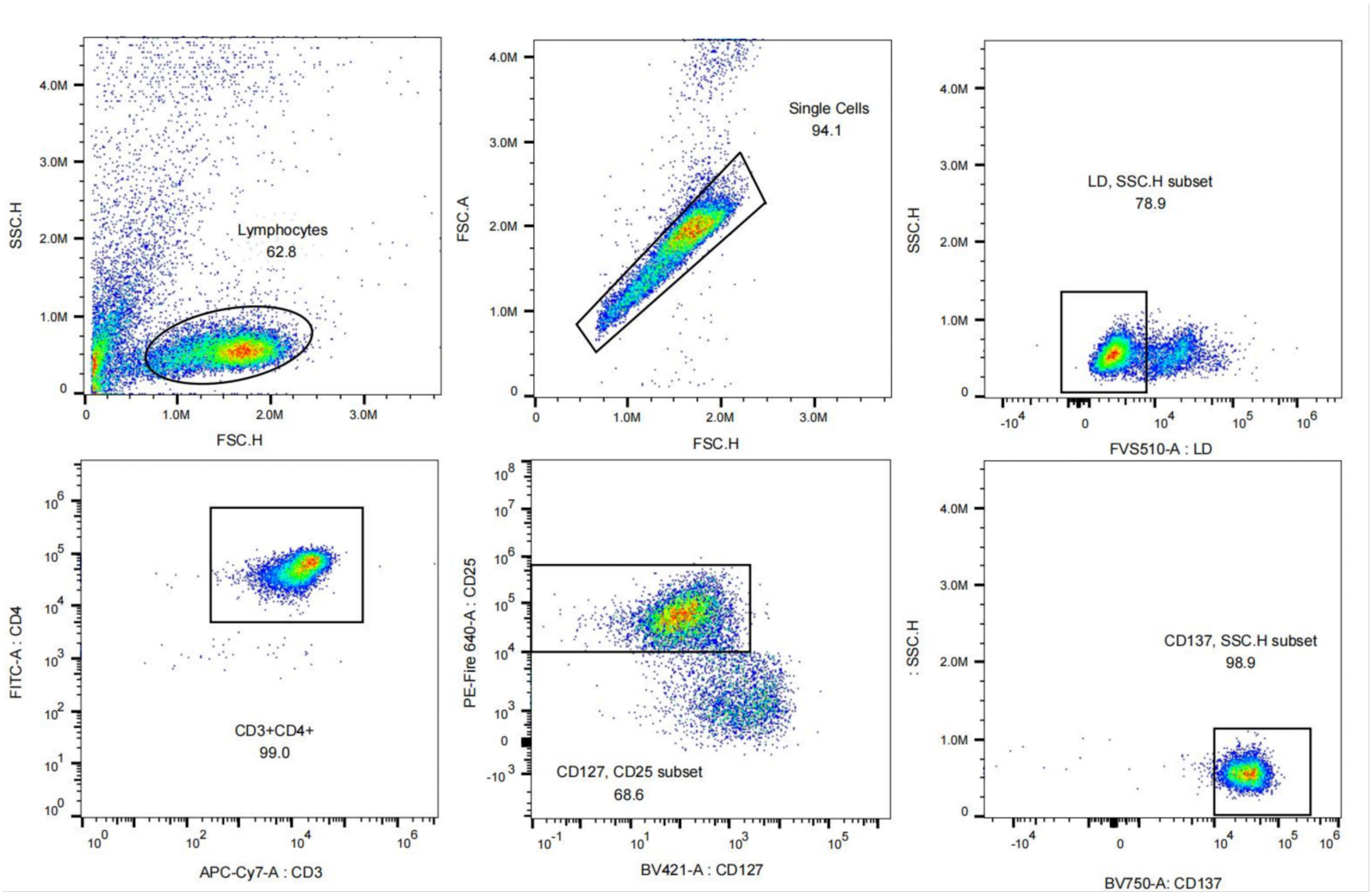

Supplementary Figure 15. Gating Strategy for Assessing Purity of Bead-Enriched CD137+ Tregs singlets and live cells were sequentially gated from FSC/SSC, followed by selection of CD3+CD4+T cells, then CD25+CD127- Tregs, and finally the CD137+population.

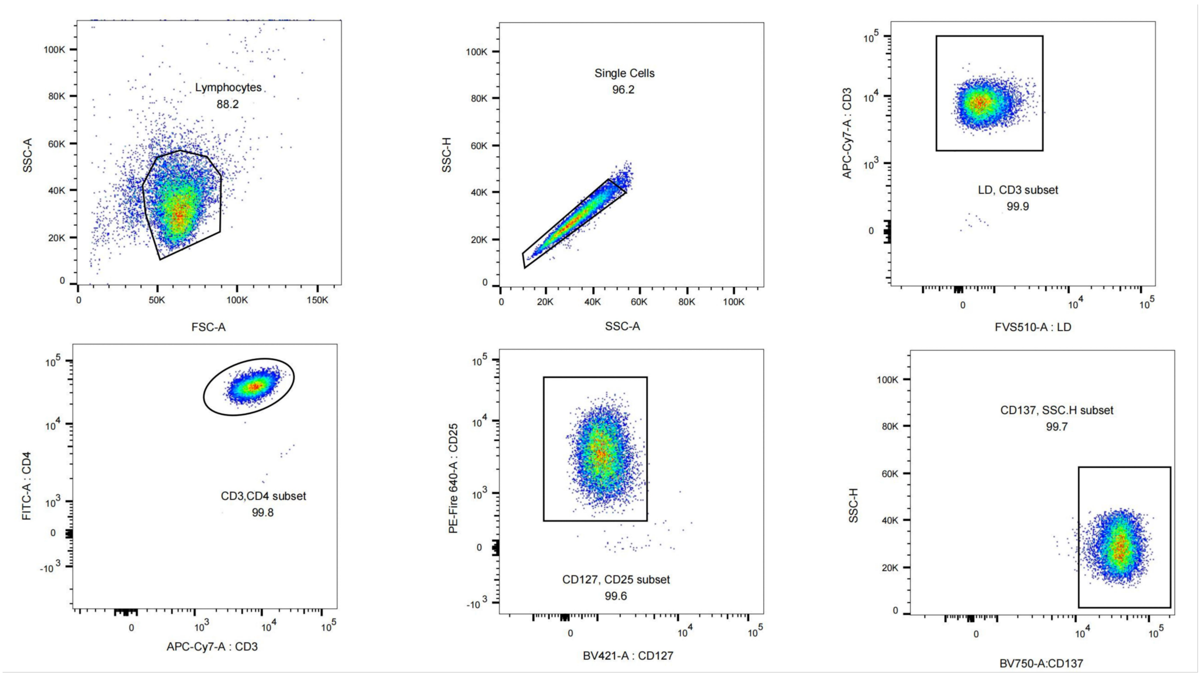

Supplementary Figure 16. Gating Strategy for Assessing Purity of Flow Cytometry-Isolated CD137+ Tregs

singlets and live cells were sequentially gated from FSC/SSC, followed by selection of CD3+CD4+T cells, then CD25+CD127- Tregs, and finally the CD137+population.

Supplementary Table 1. Clinical data from the Cohort1

	ICI-IA (n=21)		ICI-NC (n=9)		RA-CA (n=7)	
	Mean/N	SD/%	Mean/N	SD/%	Mean/N	SD/%
Male	10	47.6	4	44.4	4	57.1
Female	11	52.4	5	55.6	3	42.9
Asian ethnicity	21	100	9	100	7	100
Age	55.14	8.89	57.44	11.05	55.14	11.91
Cancer types						
CRC	4	19.00	0	0	0	0
GC	4	19.00	3	33.30	1	14.30
HCC	2	9.50	1	11.10	1	14.30
NSCLC	4	19.00	1	11.10	2	28.60
Pancreatic cancer	7	33.30	4	44.40	3	42.90
Cancer stage						
II	11	52.40	9	100.00	3	42.90
III	10	47.60	0	0	4	57.10
ICI types						
Camrelizumab	5	23.80	1	11.10	--	--
Durvalumab	3	14.30	2	22.20	--	--
Nivolumab	4	19.00	1	11.10	--	--
Pembrolizumab	5	23.80	3	33.30	--	--
Sintilimab	3	14.30	1	11.10	--	--
Toripalimab	1	4.80	1	11.10	--	--
Chemotherapy	17	81.00	7	77.80	6	85.70
Neoadjuvant_tx	4	19.00	4	44.40	2	28.60
Onset times since ICI therapy (weeks)	5.90	3.32	--	--	--	--
DMARDs tx						
Leflunomide	3	14.30	--	--	1	14.30
Methotrexate	10	47.60	--	--	6	85.70
Methotrexate+Leflunomide	3	14.30	--	--	0	0
Methotrexate+Sulfasalazine	5	23.80	--	--	0	0
Intra-articular steroid tx	16	76.20	--	--	6	85.70
Systemic steroid tx	17	81.00	--	--	3	42.90
NSAIDs tx	18	85.70	--	--	5	71.40
ANA-negative	13	61.90	--	--	4	57.10
RF-negative	21	100.00	--	--	0	0
ACPA-negative	14	66.70	--	--	0	0
OMERACT-WIPE score at onset	29.00	15.11	--	--	38.71	12.51
CRP	24.24	18.46	3.89	1.78	30.56	19.16
CDAI score at onset	23.05	9.70	--	--	19.29	6.32
CTCAE grade at onset						
1	3	14.30	--	--	--	--
2	9	42.90	--	--	--	--
3	9	42.90	--	--	--	--

ICI-IA, immune checkpoint inhibitor-inflammatory arthritis; ICI-NC, ICI-treated non-irAE cancer patients ; RA-CA, rheumatoid arthritis comorbid with cancer; N, number; SD, standard deviation; CRC, colorectal carcinoma; GC, gastric cancer; HCC, hepatocellular carcinoma; NSCLC, non-small cell lung cancer; Neoadjuvant_tx, neoadjuvant treatment before surgery; DMARDs, disease-modifying antirheumatic drugs; NSAIDs, non-steroidal anti-inflammatory drugs; ANA, anti-nuclear antibody; RF, rheumatic factor; ACPA, anti-cyclic citrullinated peptide antibodies; OMERACT-WIPE, outcome measures in rheumatoid arthritis clinical trials-MRI whole-body score for inflammation in peripheral joints and entheses; CRP, C-reactive protein; CDAI, clinical disease activity index; CTCAE, common terminology criteria for adverse events.

Supplementary Table 2. Clinical data from the Cohort2

	ICI-IA (n=23)	
	Mean/N	SD/%
Male	16	69.6
Female	7	30.4
Asian ethnicity	23	100
Age	57.70	5.77
Cancer types		
GC	23	100.00
Cancer stage		
II	7	30.40
III	14	60.90
IV	2	8.70
ICI types		
Camrelizumab	3	13.00
Durvalumab	2	8.70
Nivolumab	17	73.90
Sintilimab	1	4.30
Chemotherapy	18	78.30
Neoadjuvant_tx	23	100.00
Onset times since ICI therapy (weeks)	7.09	4.74
DMARDs		
Methotrexate	18	78.30
None	5	21.70
Intra-articular steroid	16	69.60
Systemic steroid	23	100.00
NSAIDs	5	21.70
ANA-negative	15	65.20
RF-negative	22	95.70
ACPA-negative	20	87.00
OMERACT-WIPE score at onset	36.91	12.93
CRP at onset	101.83	81.50
CDAI score at onset	21.17	8.36
FACT-G score at onset	57.65	21.35
CTCAE grade at onset		
1	2	8.70
2	11	47.80
3	10	43.50

ICI-IA, immune checkpoint inhibitor-inflammatory arthritis; N, number; SD, standard deviation; GC, Gastric cancer; Neoadjuvant_tx, Neoadjuvant ICI treatment before surgery; DMARDs, disease-modifying antirheumatic drugs; NSAIDs, non-steroidal anti-inflammatory drugs; ANA, anti-nuclear antibody; RF, rheumatic factor; ACPA, anti-cyclic citrullinated peptide antibodies; OMERACT-WIPE, outcome measures in rheumatoid arthritis clinical trials-MRI whole-body score for inflammation in peripheral joints and entheses; CRP, C-reactive protein; CDAI, clinical disease activity index; FACT-G, functional assessment of cancer therapy - general; CTCAE, common terminology criteria for adverse events.

Supplementary Table 3. Clinical data from the Cohort3

	ICI-IA (n=18)	
	Mean/N	SD/%
Male	12	66.7
Female	6	33.3
Asian ethnicity	18	100
Age	54.17	7.00
Cancer types		
GC	18	100.00
BMI	27.72	2.35
Cancer stage		
II	9	50.00
III	9	50.00
ICI types		
Camrelizumab	3	16.70
Nivolumab	12	66.70
Pembrolizumab	2	11.10
Sintilimab	1	5.60
Chemotherapy	7	38.90
Other irAE	2	11.10
Onset times since ICI therapy (weeks)	4.44	2.94
DMARDs tx		
Leflunomide	1	5.60
Methotrexate	7	38.90
Methotrexate+Leflunomide	3	16.70
Methotrexate+Sulfasalazine	2	11.10
None	5	27.80
NSAIDs tx	0	0.00
ANA-negative	11	61.10
RF-negative	16	88.90
ACPA-negative	8	44.40
OMERACT-WIPE score at onset	38.00	13.45
CRP at onset	139.38	35.09
CDAI score at onset	25.39	3.55
FACT-G score at onset	52.94	13.36
CTCAE grade at onset		
1	1	5.60
2	7	38.90
3	10	55.60

ICI-IA, immune checkpoint inhibitor-inflammatory arthritis; N, number; SD, standard deviation; BMI, body mass index; irAE, immune-related adverse events; DMARDs, disease-modifying antirheumatic drugs; NSAIDs, non-steroidal anti-inflammatory drugs; ANA, anti-nuclear antibody; RF, rheumatic factor; ACPA, anti-cyclic citrullinated peptide antibodies; OMERACT-WIPE, outcome measures in rheumatoid arthritis clinical trials-MRI whole-body score for inflammation in peripheral joints and entheses; CRP, C-reactive protein; CDAI, clinical disease activity index; FACT-G, functional assessment of cancer therapy - general; CTCAE, common terminology criteria for adverse events.

Supplementary Table.4: Sample sheet for cohort 1

PT_NAME	dx	CASE_CON	RNAseq_blood	Abseq_blood	secretomics_blood	VDJ_blood	RNAseq_SF	Abseq_SF	secretomics_SF	VDJ_SF	Abseq_Tumor	secretomics_Tumor	VDJ_Tumor	FC_blood	SEX	AGE	CANCER
IA1	ICI-IA	CASE	Treg	CD45+	--	--	--	--	--	--	--	--	--	--	F	44	GC
IA2	ICI-IA	CASE	Treg	CD45+&CD137+/-Treg	CD137+/-Treg	--	--	--	--	--	--	--	--	--	F	68	GC
IA3	ICI-IA	CASE	Treg	CD45+&CD137+/-Treg	CD137+/-Treg	--	--	--	--	--	--	--	--	--	F	62	NSCLC
IA4	ICI-IA	CASE	Treg	CD45+&CD137+/-Treg	CD137+/-Treg	--	--	--	--	--	--	--	--	--	F	47	GC
IA5	ICI-IA	CASE	--	CD45+&CD137+/-Treg	CD137+/-Treg	--	--	--	--	--	--	--	--	--	M	57	Pancreatic
IA6	ICI-IA	CASE	--	CD137+/-Treg	CD137+/-Treg	--	--	--	--	--	--	--	--	--	F	57	NSCLC
IA7	ICI-IA	CASE	--	CD137+/-Treg	CD137+/-Treg	--	--	--	--	--	--	--	--	--	F	61	Pancreatic
IA8	ICI-IA	CASE	--	CD137+/-Treg	CD137+/-Treg	--	--	--	--	--	--	--	--	--	M	46	Pancreatic
IA9	ICI-IA	CASE	--	CD137+/-Treg	CD137+/-Treg	--	--	--	--	--	--	--	--	--	F	62	HCC
RA6	RA-CA	CON	Treg	CD45+	--	--	--	--	--	--	--	--	--	--	M	51	NSCLC
RA7	RA-CA	CON	Treg	CD45+	--	--	--	--	--	--	--	--	--	--	M	65	HCC
RA8	RA-CA	CON	Treg	CD45+	--	--	--	--	--	--	--	--	--	--	F	65	GC
RA9	RA-CA	CON	--	CD45+	--	--	--	--	--	--	--	--	--	--	F	72	NSCLC
NC15	ICI-NC	CON	--	CD45+	--	--	--	--	--	--	--	--	--	--	F	67	GC
NC16	ICI-NC	CON	Treg	CD45+	--	--	--	--	--	--	--	--	--	--	F	70	HCC
NC17	ICI-NC	CON	Treg	CD45+	--	--	--	--	--	--	--	--	--	--	M	59	NSCLC
NC18	ICI-NC	CON	Treg	CD45+	--	--	--	--	--	--	--	--	--	--	F	68	GC
NC19	ICI-NC	CON	Treg	CD45+	--	--	--	--	--	--	--	--	--	--	M	44	GC
IAPt3	ICI-IA	CASE	--	Treg	CD137+/-Treg	Treg	--	Treg	CD137+/-Treg	Treg	Treg	CD137+/-Treg	Treg	--	M	43	Pancreatic
IAPt4	ICI-IA	CASE	--	Treg	CD137+/-Treg	Treg	--	Treg	CD137+/-Treg	Treg	Treg	CD137+/-Treg	Treg	--	F	64	Pancreatic

PT_NAME	dx	CASE_CON	RNAseq_blood	Abseq_blood	secretomics_blood	VDJ_blood	RNAseq_SF	Abseq_SF	secretomics_SF	VDJ_SF	Abseq_Tumor	secretomics_Tumor	VDJ_Tumor	FC_blood	SEX	AGE	CANCER
IAPt5	ICI-IA	CASE	--	Treg	CD137+/-Treg	Treg	--	Treg	CD137+/-Treg	Treg	Treg	CD137+/-Treg	Treg	--	F	71	Pancreatic
IAPt6	ICI-IA	CASE	--	Treg	CD137+/-Treg	Treg	--	Treg	CD137+/-Treg	Treg	Treg	CD137+/-Treg	Treg	--	M	62	Pancreatic
RA1	RA-CA	CON	--	Treg	--	--	--	Treg	--	--	Treg	--	--	--	M	46	Pancreatic
RA2	RA-CA	CON	--	Treg	--	--	--	Treg	--	--	Treg	--	--	--	F	44	Pancreatic
RA11	RA-CA	CON	--	Treg	--	--	--	Treg	--	--	Treg	--	--	--	M	43	Pancreatic
NC7	ICI-NC	CON	--	Treg	--	--	--	Treg	--	--	Treg	--	--	--	F	64	Pancreatic
NC8	ICI-NC	CON	--	Treg	--	--	--	Treg	--	--	Treg	--	--	--	M	43	Pancreatic
NC9	ICI-NC	CON	--	Treg	--	--	--	Treg	--	--	Treg	--	--	--	F	58	Pancreatic
NC10	ICI-NC	CON	--	Treg	--	--	--	Treg	--	--	Treg	--	--	--	M	44	Pancreatic
FC1	ICI-IA	CASE	--	--	--	--	--	--	--	--	--	--	--	Treg*4(ICI-naive+preclinical+onset+followup)	F	50	CRC
FC2	ICI-IA	CASE	--	--	--	--	--	--	--	--	--	--	--	Treg*4(ICI-naive+preclinical+onset+followup)	F	46	NSCLC
FC3	ICI-IA	CASE	--	--	--	--	--	--	--	--	--	--	--	Treg*4(ICI-naive+preclinical+onset+followup)	M	56	NSCLC
FC4	ICI-IA	CASE	--	--	--	--	--	--	--	--	--	--	--	Treg*4(ICI-naive+preclinical+onset+followup)	M	50	CRC
Pt11	ICI-IA	CASE	--	Treg*3(preclinical+onset+followup)	--	Treg*3(preclinical+onset+followup)	--	Treg*3(preclinical+onset+followup)	--	Treg*3(preclinical+onset+followup)	--	--	--	--	M	62	GC
Pt12	ICI-IA	CASE	--	Treg*3(preclinical+onset+followup)	--	Treg*3(preclinical+onset+followup)	--	Treg*3(preclinical+onset+followup)	--	Treg*3(preclinical+onset+followup)	--	--	--	--	M	43	HCC
Pt13	ICI-IA	CASE	--	Treg*3(preclinical+onset+followup)	--	Treg*3(preclinical+onset+followup)	--	Treg*3(preclinical+onset+followup)	--	Treg*3(preclinical+onset+followup)	--	--	--	--	M	45	CRC
Pt14	ICI-IA	CASE	--	Treg*3(preclinical+onset+followup)	--	Treg*3(preclinical+onset+followup)	--	Treg*3(preclinical+onset+followup)	--	Treg*3(preclinical+onset+followup)	--	--	--	--	M	62	CRC

Supplementary Table.5: Sample sheet for cohort 2

ID	VDJ_blood	VDJ_SF	VDJ_Tumor	Abseq_blood	Abseq_SF	Abseq_Tumor	sex	age	CANCER
1	Treg	Treg	Treg	Treg	Treg	Treg	M	56	Gastric
2	Treg	Treg	Treg	Treg	Treg	Treg	M	67	Gastric
3	Treg	Treg	Treg	Treg	Treg	Treg	M	61	Gastric
4	Treg	Treg	Treg	Treg	Treg	Treg	F	62	Gastric
5	Treg	Treg	Treg	Treg	Treg	Treg	M	62	Gastric
6	Treg	Treg	Treg	Treg	Treg	Treg	M	60	Gastric
7	Treg	Treg	Treg	Treg	Treg	Treg	F	61	Gastric
8	Treg	Treg	Treg	Treg	Treg	Treg	M	53	Gastric
9	Treg	Treg	Treg	Treg	Treg	Treg	F	52	Gastric
10	Treg	Treg	Treg	Treg	Treg	Treg	M	55	Gastric
11	Treg	Treg	Treg	Treg	Treg	Treg	F	53	Gastric
12	Treg	Treg	Treg	Treg	Treg	Treg	M	63	Gastric
13	Treg	Treg	Treg	Treg	Treg	Treg	M	50	Gastric
14	Treg	Treg	Treg	Treg	Treg	Treg	M	53	Gastric
15	Treg	Treg	Treg	Treg	Treg	Treg	M	55	Gastric
16	Treg	Treg	Treg	Treg	Treg	Treg	F	52	Gastric
17	Treg	Treg	Treg	Treg	Treg	Treg	M	70	Gastric
18	Treg	Treg	Treg	Treg	Treg	Treg	M	61	Gastric
19	Treg	Treg	Treg	Treg	Treg	Treg	F	66	Gastric
20	Treg	Treg	Treg	Treg	Treg	Treg	M	52	Gastric
21	Treg	Treg	Treg	Treg	Treg	Treg	F	52	Gastric
22	Treg	Treg	Treg	Treg	Treg	Treg	M	60	Gastric
23	Treg	Treg	Treg	Treg	Treg	Treg	M	51	Gastric

Supplementary Table.6: Sample sheet for cohort 3

P	VDJ_blood	VDJ_SF	VDJ_Tumor	Abseq_blood	Abseq_SF	VDJ_Tumor	RNAseq_blood	RNAseq_SF	RNAseq_Tumor	sex	age	CANCER
1	Treg*2(pre&postTx)	Treg*2(pre&postTx)	Treg	Treg*2(pre&postTx)	Treg*2(pre&postTx)	Treg	--	--	--	M	50	Gastric
2	Treg*2(pre&postTx)	Treg*2(pre&postTx)	Treg	Treg*2(pre&postTx)	Treg*2(pre&postTx)	Treg	--	--	--	F	46	Gastric
3	Treg*2(pre&postTx)	Treg*2(pre&postTx)	Treg	Treg*2(pre&postTx)	Treg*2(pre&postTx)	Treg	--	--	--	F	51	Gastric
4	Treg*2(pre&postTx)	Treg*2(pre&postTx)	Treg	Treg*2(pre&postTx)	Treg*2(pre&postTx)	Treg	--	--	--	M	47	Gastric
5	Treg*2(pre&postTx)	Treg*2(pre&postTx)	Treg	Treg*2(pre&postTx)	Treg*2(pre&postTx)	Treg	--	--	--	F	56	Gastric
6	Treg*2(pre&postTx)	Treg*2(pre&postTx)	Treg	Treg*2(pre&postTx)	Treg*2(pre&postTx)	Treg	--	--	--	F	68	Gastric
7	Treg*2(pre&postTx)	Treg*2(pre&postTx)	Treg	Treg*2(pre&postTx)	Treg*2(pre&postTx)	Treg	Treg*2(pre&postTx)	Treg*2(pre&postTx)	Treg	M	54	Gastric
8	Treg*2(pre&postTx)	Treg*2(pre&postTx)	Treg	Treg*2(pre&postTx)	Treg*2(pre&postTx)	Treg	--	--	--	M	52	Gastric
9	Treg*2(pre&postTx)	Treg*2(pre&postTx)	Treg	Treg*2(pre&postTx)	Treg*2(pre&postTx)	Treg	--	--	--	M	53	Gastric
10	Treg*2(pre&postTx)	Treg*2(pre&postTx)	Treg	Treg*2(pre&postTx)	Treg*2(pre&postTx)	Treg	Treg*2(pre&postTx)	Treg*2(pre&postTx)	Treg	M	60	Gastric
11	Treg*2(pre&postTx)	Treg*2(pre&postTx)	Treg	Treg*2(pre&postTx)	Treg*2(pre&postTx)	Treg	--	--	--	F	55	Gastric
12	Treg*2(pre&postTx)	Treg*2(pre&postTx)	Treg	Treg*2(pre&postTx)	Treg*2(pre&postTx)	Treg	--	--	--	F	50	Gastric
13	Treg*2(pre&postTx)	Treg*2(pre&postTx)	Treg	Treg*2(pre&postTx)	Treg*2(pre&postTx)	Treg	Treg*2(pre&postTx)	Treg*2(pre&postTx)	Treg	M	55	Gastric
14	Treg*2(pre&postTx)	Treg*2(pre&postTx)	Treg	Treg*2(pre&postTx)	Treg*2(pre&postTx)	Treg	--	--	--	M	69	Gastric
15	Treg*2(pre&postTx)	Treg*2(pre&postTx)	Treg	Treg*2(pre&postTx)	Treg*2(pre&postTx)	Treg	--	--	--	M	43	Gastric
16	Treg*2(pre&postTx)	Treg*2(pre&postTx)	Treg	Treg*2(pre&postTx)	Treg*2(pre&postTx)	Treg	--	--	--	M	62	Gastric
17	Treg*2(pre&postTx)	Treg*2(pre&postTx)	Treg	Treg*2(pre&postTx)	Treg*2(pre&postTx)	Treg	--	--	--	M	55	Gastric
18	Treg*2(pre&postTx)	Treg*2(pre&postTx)	Treg	Treg*2(pre&postTx)	Treg*2(pre&postTx)	Treg	--	--	--	M	49	Gastric

Supplementary Table.7:Incidence of ICI-IA in Study Cohorts

Study Cohort	Participating Center(s)	Enrollment Period	Screened Cases	Confirmed Cases	Incidence
Cohort 1	Affiliated Cancer Hospital of Shantou University Medical College	Jun 2021 – May 2022	98	5	0.05
Cohort 2	Sun Yat-Sen Cancer Center & Cancer Hospital of Shantou University Medical College	Jan 2022 – Apr 2023	285	23	0.08
Cohort 3	Cancer Hospital of Shantou Univ., First Affiliated Hosp. of Zhengzhou Univ., Hainan Hosp. of PLA General Hosp.	Nov 2022 – Jul 2024	645	18	0.03

Supplementary Table.8: Single-cell surface markers used to profile the cell-surface phenotype

ID	ABSEQ_CAT
CCR7	940394
CD11a	940077
CD127	940012
CD210A	940500
CD226	940075
CD25	940009
CD3	940000
CD4	940001
CD40L	940053
HLA_I	940062
CD45	940002
CD2	940046
CD45RA	940011
CD8	940003
CTLA4	940034
CD19	940004
CD20	940016
CD27	940018
IGHD	940026
CD16	940006
CD56	940007
CD335	940064
GITR	940096
ICOS	940043
IL2RB	940232
CXCR6	940234
ITGAE	940067
TNFRSF9	940055
PD1	940015
TNFRSF4	940060
VISTA	940497
CCR2	940286

ID	ABSEQ_CAT
CD14	940005
CD64	940262
BDCA2	940282
ITGAX	940024
HLA_II	940010

Informed Consent Form for Tocilizumab Injection Treatment

Patient Name: ________ Medical Record Number: ________ Age: ________ Gender: ________

Dear Patient,

Based on your medical condition, we recommend treatment with Tocilizumab (Actemra). Before you decide whether to accept this treatment, please read the following information carefully. Your attending physician will also provide you with a detailed explanation.

I. Background and Purpose of Treatment

You are currently undergoing immune checkpoint inhibitor therapy. While these drugs fight cancer, they may also trigger an over-activation of the immune system, attacking your own normal joints and leading to severe, persistent arthritis, known as immune checkpoint inhibitor-associated arthritis. Conventional anti-inflammatory analgesics or glucocorticoids have not provided satisfactory control of your joint symptoms, or you have been unable to tolerate their side effects.
Tocilizumab is a targeted drug that works by blocking the interleukin-6 receptor to suppress excessive inflammatory responses. Although its role in cancer treatment itself is still being explored, both domestic and international clinical practice and literature reports indicate that this drug may be effective in controlling severe inflammatory arthritis triggered by immunotherapy.
The primary objectives of using Tocilizumab are: to control your joint inflammation, alleviate pain, improve joint function and quality of life, with the aim of enabling you to continue anti-tumor treatment safely.

II. Treatment Plan

Drug Name: Tocilizumab Injection.
Recommended Dosage and Course: Based on your body weight, the recommended dose is 8 mg/kg, administered via intravenous infusion every 2 weeks. The treatment duration will be determined based on the response of your inflammation and your body's tolerance, typically requiring several infusions. During the treatment period, your doctor will closely monitor your joint symptoms and inflammatory markers.

III. Potential Benefits of Treatment

To reduce joint swelling, pain, and stiffness; improve joint mobility and daily living functions; enhance quality of life; and potentially help you continue necessary anti-tumor therapy.

IV. Potential Risks and Adverse Reactions of Treatment

Despite preventive measures taken by your doctor, the following adverse reactions may occur during treatment, including but not limited to:
Increased Risk of Infection: Most commonly upper respiratory tract infections and

nasopharyngitis. The risk of serious infections may also increase.
Gastrointestinal Reactions: Nausea, abdominal pain, mouth ulcers, etc.
Impact on Liver Function: May cause transient elevation of liver enzymes.
Impact on Hematologic System: May lead to decreased neutrophil and platelet counts.
Infusion Reactions: Headache, rash, itching, blood pressure fluctuations, etc., may occur during infusion.
Other Potential Risks: Intestinal perforation, increased blood lipids, skin reactions, etc.
Special Note: This drug is used to manage adverse reactions related to immunotherapy and is not currently used directly as an anti-tumor agent. Its long-term effects on your underlying tumor are not yet fully understood.

V. Alternative Treatment Options

Other treatment options you may consider include:
Switching to other types of immunosuppressants.
Continuing or adjusting the dosage and administration of glucocorticoids.
Pausing or discontinuing the current immune checkpoint inhibitor therapy.
You have the right to understand the pros and cons of all available options and to discuss them with your attending physician.

VI. Patient Declaration and Consent

The attending physician has explained to me in detail my condition, the necessity, purpose, plan, expected benefits, potential risks, and alternative options of Tocilizumab treatment.
I have read and understood the above content, and all my questions have been patiently answered by the doctor. I understand that this treatment plan is a choice for my current specific condition. I understand that during the treatment period, I need to cooperate with the doctor for regular check-ups, including blood tests, liver and kidney function tests, inflammatory markers, and necessary imaging studies. I voluntarily consent to receive Tocilizumab treatment and accept the associated risks.

VII. Signatures

This consent form is made in duplicate, with one copy held by each party.

Patient (or Legal Representative) Signature: ________
Date: //____
Contact Phone Number: ________

Attending Physician Signature: ________
Date: //____

TCR_clonotype	SAMPLE	EXPAND	TRBV	TRBD	TRBJ	TRAV	TRAJ
TGTGCAGCAAGCGCGATCAGGGGAGGAAAGCTTATCTTC_TGTGCCAGCAGCTACAAGCGGGTTCTCACAGATACGCAGTATTTT	B_4	0.072096863	TRBV13	TRBD2	TRBJ2-3	TRAV29DV5	TRAJ23
TGTGCAGCAAGGGGTGGTGCTACAAACAAGCTCATCTTT_TGTGCCAGCAGCGCCGACTGGCTTGGGAACAATGAGCAGTTCTTC	B_4	0.063841497	TRBV7-9	0	TRBJ2-1	TRAV29DV5	TRAJ32
TGTGCAGCAATCCCTAACCAGGGAGGAAAGCTTATCTTC_TGTGCCAGCAAGGGAAGCGGAATCTCCTACAATGAGCAGTTCTTC	B_4	0.088057237	TRBV13	TRBD2	TRBJ2-1	TRAV29DV5	TRAJ23
TGTGCAGCGGACGATTCAGGAGGAGGGGCGGACGGACTCACCTTT_TGTGCCAGCAGCTTATCGGGGGCACGGAGACCCAGTACTTC	B_6	0.040637013	TRBV13	TRBD1	TRBJ2-5	TRAV29DV5	TRAJ45
TGTGCAGCAAGCGAGGATTATGGAGGAAGCCAAGGAAATCTCATCTTT_TGTGCCAGCAGCCTATCTGGAGAGACCCAGTACTTC	B_3	0.036702428	TRBV13	0	TRBJ2-5	TRAV29DV5	TRAJ42
TGTGCTGTGAGTGATTGGGCCGGCACTGCCAGTAAACTCACCTTT_TGTGCCAGCAGCTTAGGCCAGGGGGCCAATTCACCCCTCCACTTT	B_3	0.033314512	TRBV13	TRBD1	TRBJ1-6	TRAV8-4	TRAJ44
TGTGCAGCATGCGAGAGAACCTCAGGAACCTACAAATACATCTTT_TGTGCCAGCACCCAGGGGGGCAGCTTGGAGACCCAGTACTTC	B_6	0.029654036	TRBV13	TRBD1	TRBJ2-5	TRAV29DV5	TRAJ40
TGTGCAGCCCCCCCGGGGGGAGGTAGCAACTATAAACTGACATTT_TGTGCCAGCAGCTTTCGTCGGCCAACCGGGGAGCTGTTTTTT	B_5	0.028856243	TRBV13	0	TRBJ2-2	TRAV29DV5	TRAJ53
TGTGCAGCAAGCGCGCTGGGAAAACCTCCTACGACAAGGTGATATTT_TGTGCCAGCAGCTTCGACAGTTCTGGAAACACCATATATTTT	B_3	0.033879164	TRBV13	0	TRBJ1-3	TRAV29DV5	TRAJ50
TGTGCAGCAAGTGGGGATACCGGCACTGCCAGTAAACTCACCTTT_TGTGCCAGCAGCCGTTGGGGCGATGAGCAGTTCTTC	B_3	0.025974026	TRBV13	TRBD1	TRBJ2-1	TRAV29DV5	TRAJ44
TGTGCAGTGCATTATTATGGAGGAAGCCAAGGAAATCTCATCTTT_TGTGCCAGCAGCTTGACAGCCAATCAGCCCCAGCATTTT	B_5	0.013641133	TRBV13	TRBD1	TRBJ1-5	TRAV29DV5	TRAJ42
TGTGCAGCAAGCGGGGTCTATGGAGGAAGCCAAGGAAATCTCATCTTT_TGTGCCAGCAGCCCCGCCGTCGGGAGGGCAGACAATGAGCAGTTCTTC	B_3	0.029361942	TRBV13	TRBD2	TRBJ2-1	TRAV29DV5	TRAJ42
TGTGCAGCACATGCTGGTGGTACTAGCTATGGAAAGCTGACATTT_TGTGCCAGCAGCGCAGGGGTAGCCGAGGGCTATGGCTACACCTTC	B_6	0.01867106	TRBV9	TRBD2	TRBJ1-2	TRAV29DV5	TRAJ52
TGTGCAGCAAGTGCCATTAGTGGAGGTAGCAACTATAAACTGACATTT_TGTGCCAGCAGCCGGGGACAGGGGTTCTACGAGCAGTACTTC	B_3	0.021456804	TRBV13	TRBD1	TRBJ2-7	TRAV29DV5	TRAJ53
TGTGCAGCCGGGATCCTTAACACCAATGCAGGCAAATCAACCTTT_TGTGCCAGCAGCAAAGGACAGGGGAGTGTCGGGGAGCTGTTTTTT	B_6	0.034596376	TRBV13	TRBD1	TRBJ2-2	TRAV29DV5	TRAJ27
TGTGCAGCACCCCTCAATAATAATGCAGGCAACATGCTCACCTTT_TGTGCCAGCAGCTTAGGAGCGGGATTTAATGAGCAGTTCTTC	B_6	0.029654036	TRBV13	TRBD2	TRBJ2-1	TRAV29DV5	TRAJ39
TGTGCAGCCCTTAATAGTGGAGGTAGCAACTATAAACTGACATTT_TGTGCCAGCAGCTTATACAGGGACTACGAGCAGTACTTC	B_5	0.017838405	TRBV13	TRBD1	TRBJ2-7	TRAV29DV5	TRAJ53
TGTGCAGCCATTTATTATGGAGGAAGCCAAGGAAATCTCATCTTT_TGTGCCAGCAGGGGCGAGGGGACGAGCTCCTACGAGCAGTACTTC	B_6	0.032948929	TRBV13	TRBD1	TRBJ2-7	TRAV29DV5	TRAJ42
TGTGCTGTGAGTGATTCTAGGAGAGGAACCTACAAATACATCTTT_TGTGCCAGCAGCACCCAGGACAGCTACGAGCAGTACTTC	B_3	0.020892151	TRBV13	TRBD1	TRBJ2-7	TRAV8-6	TRAJ40
TGTGCAGCAAGCGATTTAGAGGGTGCCAGACTCATGTTT_TGTGCCAGCCGTGGCGGGGGCCTGTCCGGGGAGCTGTTTTTT	B_4	0.020363236	TRBV13	TRBD1	TRBJ2-2	TRAV29DV5	TRAJ31
TGTGCTGTGAGTGGGAGGGAAACCAGTGGCTCTAGGTTGACCTTT_TGTGCCAGCAGCTTAAAACTAGCGGCCTCCCCGGTGGAGCAGTTCTTC	B_3	0.002258611	TRBV13	TRBD2	TRBJ2-1	TRAV8-4	TRAJ58
TGTGCAGCCCCAGCAAGGAGCTGGCAACAACCGTAAGCTGATTTGG_TGTGCCAGCAGCGCAGCGGGGGGTTCTAGTCAGCCCCAGCATTTT	B_6	0.024711697	TRBV2	TRBD1	TRBJ1-5	TRAV29DV5	TRAJ38
TGTGCCGCAAGCGCCTCTGGGGCTGGGAGTTACCAACTCACGTTC_TGTGCCAGCAGCTCGGGACAGGGAAACACAGATACGCAGTATTTT	B_4	0.019262521	TRBV13	TRBD1	TRBJ2-3	TRAV29DV5	TRAJ28
TGTGCAGCCCAGCAAGGAGCTGGCAACACCCGTAAGCTGATTTGG_TGTGCCAGCAGCTTGCGAGACAATGAGCAGTTCTTC	B_5	0.017838405	TRBV13	0	TRBJ2-1	TRAV29DV5	TRAJ38
TGTGTGGTGAACAGGTATAACCAGGGAGGAAAGCTTATCTTC_TGTGCCAGCAGTTTCGGCAGTCCGGAGACCCAGTACTTC	B_2	0.017828201	TRBV12-4	TRBD2	TRBJ2-5	TRAV12-1	TRAJ23
TGTGCAGGAGCCTCAAGAACTGACAGCTGGGGGAAATTGCAGTTT_TGTGCCAGCAGTGAAACGACTGAGGCCTACAATGAGCAGTTCTTC	B_1	0.031147541	TRBV6-1	0	TRBJ2-1	TRAV27	TRAJ24
TGTGCTGTGAGGCCCAGCACCAATGCAGGCAAATCAACCTTT_TGCAGTGCGACTAGCGGGGCGACGCCGCAAGAGACCCAGTACTTC	B_5	0.029905561	TRBV20-1	TRBD2	TRBJ2-5	TRAV21	TRAJ27
TGTGCTTTCATGAAACCGAACACCGGTAACCAGTTCTATTTT_TGTGCCAGCAGCTCTAGCGGGACCCTAGCATATGAGCAGTTCTTC	B_2	0.016747704	TRBV7-2	TRBD1	TRBJ2-1	TRAV38-1	TRAJ49
TGTGCTGCGCCCGCGAACACCGGTAACCAGTTCTATTTT_TGTGCCAGCAGTGAACAGGGGATCAGCACAGATACGCAGTATTTT	B_1	0.019672131	TRBV12-3	TRBD1	TRBJ2-3	TRAV21	TRAJ49
TGTGCTGTGGGTGTACGACAGGCAGGAACTGCTCTGATCTTT_TGCGCCAGCAGCGTACCGGGACAGAAAAAAGAGACCCAGTACTTC	SF_4	0.063133281	TRBV5-1	TRBD1	TRBJ2-5	TRAV8-3	TRAJ15
TGTCCCATCCCACCCACTGGCAACACAGGCAAACTAATCTTT_TGTGCCAGCAGCTTGTTACAGTTTAGCCAAAACATTCAGTACTTC	B_2	0.01458671	TRBV5-6	0	TRBJ2-4	TRAV29DV5	TRAJ37
TGTGCAGAGTCCCCGGGGGGGGCTGGGAGTTACCAACTCACTTTC_TGTGCCAGCAGCTCGACTAGCGGAGCGTCGATGAGCAGTTCTTC	B_1	0.015300546	TRBV28	TRBD2	TRBJ2-1	TRAV13-2	TRAJ28
TGTGCTACGGGGGAAGGACTGGGAGCCCAGAAGCTGGTATTT_TGTGCCAGCAGTAGTATTTCTCCAGATAATTCACCCCTCCACTTT	SF_6	0.060289389	TRBV19	0	TRBJ1-6	TRAV17	TRAJ54
TGTGCTACGGGCCCTTATACCGGCACTGCCAGTAAACTCACCTTT_TGCAGTGCTGGTCCGGGACTGCCAGGATGGGAGACCCAGTACTTC	B_5	0.017838405	TRBV20-1	TRBD2	TRBJ2-5	TRAV17	TRAJ44
TGTGCTGTGGGGCCTAAAAATTCCGGGTATGCACTCAACTTC_TGCGCCAGCAGCTTGGACGGGAGGGCCTACGAGACCCAGTACTTC	B_4	0.006604293	TRBV5-1	TRBD2	TRBJ2-5	TRAV36DV7	TRAJ41
TGTGCTGTGAGACTCGGCTATAACGACTACAAGCTCAGCTTT_TGCAGTGCTAGAGTGCAATCGGGACAAGGAGGCGGCTACACCTTC	B_5	0.01521511	TRBV20-1	TRBD1	TRBJ1-2	TRAV3	TRAJ20
TGTGCAGCCACCAATAGTGGAGGTAGCAACTATAAACTGACATTT_TGTGCCAGCAGCGCGGGGCGGGTCCCCACAGATACGCAGTATTTT	B_5	0.012067156	TRBV9	TRBD1	TRBJ2-3	TRAV29DV5	TRAJ53
TGTGCAGCTAGGGGTCGAGAAACCAGTGGCTCTAGGTTGACCTTT_TGTGCCAGCAGCGTAGCTGGGACTAGCGGAGGGTTTCAAGAGACCCAGTACTTC	B_5	0.002098636	TRBV9	TRBD2	TRBJ2-5	TRAV29DV5	TRAJ58
TGTGCTGTGGAACTTACCAAAGCTGCAGGCAACAAGCTAACTTTT_TGTGCCTGGAGTGCTCCACATGGGACGGGAGGCGAGCAGTACTTC	B_10	0.025900901	TRBV30	TRBD2	TRBJ2-7	TRAV22	TRAJ17
TGTGCAGAGTTCAATAATGCAGGCAACATGCTCACCTTT_TGTGCCAGCAGCTTAGGACCCCTAAGCACAGATACGCAGTATTTT	B_2	0.024851432	TRBV7-2	TRBD1	TRBJ2-3	TRAV5	TRAJ39
TGTGCAGAGGACCGAGGTAGCAACTATAAACTGACATTT_TGTGCCAGCAGCTTAACCCTAGCGGAGCAGAGACCCAGTACTTC	B_2	0.024311183	TRBV7-3	TRBD2	TRBJ2-5	TRAV13-2	TRAJ53
TGTGTGGTGACCTTCGATACTGGAGGCTTCAAAACTATCTTT_TGTGCCAGCAGCTTTGGTGTTAGCGGGAGCGATGAGCAGTTCTTC	B_2	0.024311183	TRBV11-2	TRBD2	TRBJ2-1	TRAV12-1	TRAJ9
TGTGCCCTGAATTATGGAGGAAGCCAAGGAAATCTCATCTTT_TGTGCCAGCTCATCGTTTCCAGGGGGCTCTGAGCAGTTCTTC	B_6	0.007688083	TRBV18	TRBD1	TRBJ2-1	TRAV8-4	TRAJ42
TGTGCAGAGACCTTGATGAACAGAGATGACAAGATCATCTTT_TGTGCCAGCAGCCCCCAAGGGGTCGGAAACACCATATATTTT	B_8	0.017582418	TRBV12-3	TRBD1	TRBJ1-3	TRAV13-2	TRAJ30
TGCATCGACGCACCAGGAAATAATGCAGGCAACATGCTCACCTTT_TGTGCCAGCAGCTATCAAGCGGGAATCACAGATACGCAGTATTTT	B_2	0.021609941	TRBV7-2	TRBD2	TRBJ2-3	TRAV26-1	TRAJ39
TGTGCAGCAAGCCCAGGAGGAGGTGCTGACGGACTCACCTTT_TGTGCCACCAGCAGAGAGGTCCTCTTCACAGATACGCAGTATTTT	B_11	0.009049774	TRBV15	0	TRBJ2-3	TRAV23DV6	TRAJ45
TGTGCTTTCATGAAGCAAGCCAGTGGCTCTAGGTTGACCTTT_TGTGCCAGCAGCTTAGGCTCTAGCGGGCAAGAGACCCAGTACTTC	B_2	0.008643976	TRBV7-6	TRBD2	TRBJ2-5	TRAV38-1	TRAJ58
TGTGCTGTGCAGGGCGGTAGCAACACAGGCAAACTAATCTTT_TGCGCCAGCAGCTCGTTTTTTGGGGGCACCGGGGAGCTGTTTTTT	SF_4	0.02650039	TRBV5-1	TRBD1	TRBJ2-2	TRAV20	TRAJ37
TGTGCAGCAAGCGCGCGTGGCTTTGGGAATGTGCTGCATTGC_TGTGCCAGCAGCCCGGGACAGAACAGCTATGGCTACACCTTC	B_8	0.008791209	TRBV6-5	TRBD1	TRBJ1-2	TRAV29DV5	TRAJ35
TGTGCAGAGAATTCCTTCTCAGGAACCTACAAATACATCTTT_TGCAGCGTTGGAAACCAGGGTACCTACGAGCAGTACTTC	B_9	0.006802721	TRBV29-1	TRBD1	TRBJ2-7	TRAV13-2	TRAJ40
TGTGCTGTGAGAGACGGGCCTGGGGCAAACAACCTCTTCTTT_TGCAGCGTTGTGGCTTGGCAGGGGAGGTTGTGCGAGCAGTACTTC	B_5	0.001573977	TRBV29-1	TRBD1	TRBJ2-7	TRAV3	TRAJ36
TGTGCAGCCGTGAATTATGGAGGAAGCCAAGGAAATCTCATCTTT_TGTGCCAGCAGCGCACTAGGGACAGATCAAGAGACCCAGTACTTC	B_5	0.001049318	TRBV9	TRBD1	TRBJ2-5	TRAV29DV5	TRAJ42
TGTGCAATGAGAGCAATGAATTCAGGAAACACACCTCTTGTCTTT_TGTGCCAGCAGTTTAGCTGTTAGACTGAGGAATGAGCAGTTCTTC	B_1	0.002185792	TRBV28	0	TRBJ2-1	TRAV14DV4	TRAJ29
TGTGCAATGAGCCGGGATAACTATGGTCAGAATTTTGTCTTT_TGTGCCAGCAGCATTACAGGGTCGAACTATGGCTACACCTTC	B_9	0.011337868	TRBV6-1	TRBD1	TRBJ1-2	TRAV12-3	TRAJ26
TGCATCGACGCACCAGGAAATAATGCAGGCAACATGCTCACCTTT_TGTGCCAGCAGCCTTGGGATGGGGCTGAGGACTGAAGCTTTCTTT	B_2	0.006482982	TRBV14	TRBD1	TRBJ1-1	TRAV26-1	TRAJ39
TGCCTCGTGGGTGACATTCGGAATAACAATGCCAGACTCATGTTT_TGTGCCAGCAGTTACGCAGACGGCAGAACCTACGAGCAGTACTTC	B_1	0.013661202	TRBV6-6	0	TRBJ2-7	TRAV4	TRAJ31
TGTGCAATGAGAGAGGGCGGTAGCAATAACAATGACATGCGCTTT_TGTGCCAGCAGTTACTCGAGCGCTATCGGCTACGAGCAGTACTTC	B_1	0.013661202	TRBV6-5	TRBD2	TRBJ2-7	TRAV14DV4	TRAJ43
TGTGCAATGAGAGGGTCGACTGACAGCTGGGGGAAATTGCAGTTT_TGTGCCAGCAGTGCAGCGTCGGATTCCGGGCAGCCCCAGCATTTT	B_1	0.001639344	TRBV6-1	TRBD1	TRBJ1-5	TRAV14DV4	TRAJ24
TGTGCAGCAAGCGAGTCGGACTTTGGAAATGAGAAATTAACCTTT_TGTGCCAGCAGCTTAGCCCAGGGGAGAATCGAAGAGACCCAGTACTTC	B_1	0.007650273	TRBV13	TRBD1	TRBJ2-5	TRAV29DV5	TRAJ48
TGTGCAGCTTCAGGAGGAGGTGCTGACGGACTCACCTTT_TGTGCCAGCAGTTTGAGCGGGGTCAGCTCCTACGAGCAGTACTTC	B_1	0.013114754	TRBV28	TRBD2	TRBJ2-7	TRAV13-1	TRAJ45
TGTGCTATCCGGAAGAGGGGAGGAACCTACAAATACATCTTT_TGTGCCAGCACCCACAGGGCGGACCCTACGAGCAGTACTTC	B_6	0.007688083	TRBV7-2	TRBD1	TRBJ2-7	TRAV8-5	TRAJ40
TGCCTCGTGGGTGACCTGGACACGGGCAGGAGAGCACTTACTTTT_TGTGCCAGCAGTGCGTTTAGGGACACCAACTATGGCTACACCTTC	B_1	0.006010929	TRBV2	TRBD1	TRBJ1-2	TRAV4	TRAJ5
TGTGCTGTGAGTCAAACTGGGGCAAACAACCTCTTCTTT_TGTGCCAGCAGTTACGCGGTAGGGTCAGCAGATACGCAGTATTTT	B_1	0.012021858	TRBV6-6	TRBD2	TRBJ2-3	TRAV8-4	TRAJ36
TGTGCAGAGATGGCGGGGGCCCAGGCAGGAACTGCTCTGATCTTT_TGTGCCAGCAGTTGGAACCCTTCCCGACGGAACATTCAGTACTTC	T_1	0.012750455	TRBV2	TRBD1	TRBJ2-4	TRAV13-2	TRAJ15
TGTGCAGAGTCATATTCAGGAGGAGGTGCTGACGGACTCACCTTT_TGTGCCAGCAGTGAAGTCGGGGGGCTATCGGGGGAGCTGTTTTTT	B_1	0.01147541	TRBV6-1	TRBD1	TRBJ2-2	TRAV13-2	TRAJ45
TGCATCGTCAGAGTCTCCTTTGGAAATGAGAAATTAACCTTT_TGTGCCAGCAGGGGACTAGCGGGCACAGATACGCAGTATTTT	B_9	0.005668934	TRBV27	TRBD2	TRBJ2-3	TRAV26-1	TRAJ48
TGTGCTGTGCTAAGTGGAGGTAGCAACTATAAACTGACATTT_TGCGCCAGCAGCTTGGAGGGACAGGTCACAGATACGCAGTATTTT	SF_4	0.014809041	TRBV5-1	TRBD1	TRBJ2-3	TRAV20	TRAJ53
TGCATCGTCAGAGTCTCCTTTGGGCAGGGAGGAAAGCTTATCTTC_TGTGCCAGCAGCCTGACTAGCGGGGGACTCTACGAGCAGTACTTC	B_2	0.002701243	TRBV28	TRBD2	TRBJ2-7	TRAV26-1	TRAJ23
TGTGCTGTGAGTGCCGGTTCGAACCAGGGAGGAAAGCTTATCTTC_TGCGCCAGCAGCTATACGACCGGGACAGGGGAGGAGACCCAGTACTTC	B_10	0.004504505	TRBV5-1	TRBD1	TRBJ2-5	TRAV8-4	TRAJ23
TGTGCTGGGCGAGTAGCTTCTGGGGGTTACCAGAAAGTTACCTTT_TGCGCCAGCAGCCAAGAGGGTAGCGGGAGTATTGGTGAGCAGTTCTTC	B_10	0.002252252	TRBV4-1	TRBD2	TRBJ2-1	TRAV35	TRAJ13
TGTGCAATGAGAGAGGGTCCCGGCACTGCCAGTAAACTCACCTTT_TGTGCCAGCAGTGCTTCCCCTAGCGGGAGGGGAGAGCAGTACTTC	T_1	0.007893139	TRBV25-1	TRBD2	TRBJ2-7	TRAV14DV4	TRAJ44
TGTGCTACGGGGTCCTCCTCAGGAACCTACAAATACATCTTT_TGTGCCAGCGGGAACAGCGTGGGGGTTTACGAGCAGTACTTC	T_6	0.00843608	TRBV7-2	TRBD1	TRBJ2-7	TRAV17	TRAJ40
TGTGCCGTGACCCCTACAGCAGTGCTTCCAAGATAATCTTT_TGTGCCACCTTCATAGGTGGGGTGAGCTACGAGCAGTACTTC	B_6	0.001647446	TRBV10-3	0	TRBJ2-7	TRAV8-1	TRAJ3
TGTGCTACCCCCTACTCTGGGGCTGGGAGTTACCAACTCACTTTC_TGTGCCTGGAGTAGAGGAAACAGCTTCGATCAGCCCCAGCATTTT	B_10	0.005630631	TRBV30	0	TRBJ1-5	TRAV17	TRAJ28
TGTGCTGTGAGAGGGCCGGGGAACCAGGGAGGAAAGCTTATCTTC_TGCAGCGTTCGGGGGGCCAGTAGCGGGAGGGCCTACGAGCAGTACTTC	B_10	0.003378378	TRBV29-1	TRBD2	TRBJ2-7	TRAV3	TRAJ23
TGTGACTTAACCTTGAGTAACCAGGGAGGAAAGCTTATCTTC_TGCGCCAGCAGCTTGGTAGGACCGGGCCAGCCCCAGCATTTT	B_9	0.001133787	TRBV5-1	TRBD1	TRBJ1-5	TRAV30	TRAJ23
TGTGCAGCAAGTGGGTATTCAGGAGGAGGTGCTGACGGACTCACCTTT_TGCGCCAGCAGCTTGGCCGGCGGGCCCTCCTACGAGCAGTACTTC	SF_4	0.007794232	TRBV5-1	TRBD2	TRBJ2-7	TRAV29DV5	TRAJ45
TGTGCTACGAGGGTCAACCAGGCAGGAACTGCTCTGATCTTT_TGTGCCACCTCCACCGGGACTAGCGGGAGGAATCAGTACTTC	T_6	0.006489293	TRBV6-1	TRBD2	TRBJ2-5	TRAV17	TRAJ15
TGTGCTGTGAAGCCCACCTCAGGAACCTACAAATACATCTTT_TGTGCCGCCAGAAGGACTGCGAGCACAGATACGCAGTATTTT	T_5	0.006361323	TRBV30	0	TRBJ2-3	TRAV22	TRAJ40
TGTGCTGTGAGAGACCCCGGAGGAAGCCAAGGAAATCTCATCTTT_TGTGCCTGGAGGCAGTCCGGAGGCAGACAAGAGACCCAGTACTTC	B_10	0.003378378	TRBV30	TRBD2	TRBJ2-5	TRAV3	TRAJ42
TGTGCCGTTACCCTCTCAAATTCCGGGTATGCACTCAACTTC_TGTGCCAGCACCCCTGCCCGCGGGAGCTACGAGCAGTACTTC	B_6	0.000549149	TRBV12-3	TRBD1	TRBJ2-7	TRAV12-2	TRAJ41
TGTGCTACGGTCGCTGGTGGTACTAGCTATGGAAAGCTGACATTT_TGCGCCAGCAGCCAACGCTTAGTGAACCTCCACAATGAGCAGTTCTTC	B_10	0.001126126	TRBV4-1	0	TRBJ2-1	TRAV17	TRAJ52
TGTGCTGGGCAGTTAATTCAGGGAGCCCAGAAGCTGGTATTT_TGTGCCACCAGCCCCCGGACAGGGTCGGGCATTCAGTACTTC	B_6	0.000549149	TRBV15	TRBD1	TRBJ2-4	TRAV35	TRAJ54
TGTGCTGTGAAGCTCACGGGAGGAGGAAACAAACTCACCTTT_TGCAGTGCTAGGGGACTCTTGCGGGAGACTACTACGCAGTATTTT	B_5	0.001049318	TRBV20-1	TRBD2	TRBJ2-3	TRAV3	TRAJ10
TGTGCTGTGAGAGCCTCTGGTTCTGCAAGGCAACTGACCTTT_TGTGCCATCAGTGATGGACAGGATACAGATACGCAGTATTTT	T_5	0.005725191	TRBV10-3	TRBD1	TRBJ2-3	TRAV3	TRAJ22
TGCGCTGTGAGAGATCCGTATAACCAGGGAGGAAAGCTTATCTTC_TGTGCCAGCAGTCCCTTCGGGAGGGTCTCTCCCGAGCAGTACTTC	T_1	0.004857316	TRBV6-1	TRBD2	TRBJ2-7	TRAV1-1	TRAJ23
TGTGCAATGAGAGGGCTTACTGGAGGCTTCAAAACTATCTTT_TGTGCCAGCAGCGCCCGGGTCAGGGACAATGAGCAGTTCTTC	B_9	0.001133787	TRBV9	TRBD1	TRBJ2-1	TRAV14DV4	TRAJ9
[illegible]	[illegible]	[illegible]	[illegible]	[illegible]	[illegible]	[illegible]	[illegible]
